# In Vitro and Clinical Dermatological Effects of *Rubus crataegifolius* Fruit Extracts on Human Fibroblasts and Keratinocytes

**DOI:** 10.3390/ph19091451

**Published:** 2026-09-13

**Authors:** Yeong Eun Park, Dong Yun Kim, Je Seon Park, Seyoung Lee, Youmie Park, Taehee Kang, Min Chul Park

**Affiliations:** 1 Department of Pharmacy, Inje University, Gimhae 50834, Republic of Korea; yepark@oasis.inje.ac.kr (Y.E.P.); kdy9903@gmail.com (D.Y.K.); dolphin3922@naver.com (J.S.P.); tpdud0606@naver.com (S.L.); youmiep@inje.ac.kr (Y.P.); 2 Inje Institute of Pharmaceutical Sciences and Research, Inje University, Gimhae 50834, Republic of Korea; 3 Department of Bio Life Sciences, Shingu College, Seongnam-si 13174, Republic of Korea; thkang@shingu.ac.kr

**Keywords:** *Rubus crataegifolius* fruit extract, dermatological effects, anti-aging, hydration, LC-ESI-TOF-MS analysis

## Abstract

**Background/Objectives**: *Rubus crataegifolius* Bunge (Rosaceae, RC), a wild raspberry native to East Asia, has been investigated for the biological properties of its leaves and roots, while the dermatological potential of its fruit remains largely unexplored. This research evaluated the dermatological effects of RC fruit extracts, focusing on their potential for skin regeneration and anti-aging applications. **Methods**: RC fruit extracts were prepared by ultrasound-assisted extraction (UAE) and maceration extraction (ME) with distilled water or 70% ethanol as solvents. Phytochemical profiling was performed by liquid chromatography–electrospray ionization–time-of-flight mass spectrometry (LC-ESI-TOF-MS). Dermatological activities were evaluated using fibroblast and keratinocyte proliferation, collagen synthesis, wound healing, and gene expression assays. Safety was assessed by cytotoxicity and inflammation assays. Skin hydration was evaluated in a human application test. **Results**: Various phytochemicals, including flavonoids, phenolic acids, organic acids, and terpenoids, were putatively annotated by LC-ESI-TOF-MS in the RC fruit extracts. Ethanol extracts exhibited higher flavonoid content and antioxidant activity; however, dermatological activities were not correlated with antioxidant activity. UAE-derived extracts enhanced fibroblast proliferation. All extracts promoted collagen synthesis, with ME-EtOH showing the highest increase. In wound-healing assays, ME-DW extracts promoted fibroblast wound closure, while UAE-DW enhanced keratinocyte migration. In keratinocytes, UAE-DW specifically upregulated *HAS1* and *HAS2* mRNA expression. Clinically, UAE-DW extract produced a significant increase in skin hydration and moisture distribution in healthy volunteers. **Conclusions**: RC fruit extracts exhibit biological activities, including enhanced fibroblast proliferation, collagen synthesis, wound healing, and skin hydration.

## 1. Introduction

Aging is a multifactorial process that impacts various organs in the body. The skin, which is continuously exposed to environmental stress such as chemicals and UV radiation, undergoes progressive age-related changes that lead to structural and functional alterations, including reduced collagen content, impaired barrier integrity, dryness, and wrinkle formation [1,2]. The causes of skin aging can be divided into endogenous and exogenous factors. Endogenous factors are primarily associated with genetics and changes in cellular metabolism, hormones, and metabolic processes over time, while exogenous factors include ultraviolet, ionizing radiation, chemicals, and pollution [3,4]. These factors, especially exogenous ones, induce macromolecular structural alterations and associated functional impairments in the skin through mechanisms such as oxidative stress, inflammation, and metabolic disturbances [5]. To protect against or reverse skin aging, recent research has focused on identifying anti-aging agents from natural products, including extracellular vesicles, peptides, and phytochemicals [6,7,8].

Natural product-derived agents have been widely used in dermatology for anti-aging applications. Due to low toxicity, dermatological efficacy, broad applicability, and cost-effectiveness, plant extracts are the most extensively used natural product-derived agents in cosmeceuticals [9]. Plant extracts contain diverse phytochemicals, especially flavonoids. Flavonoid-rich extracts exhibit antioxidant activity, enabling them to neutralize oxidative stress, a key factor in aging [8,10]. Certain phytochemicals contribute to skin homeostasis by mitigating oxidative stress, preserving structural integrity, and supporting biological processes involved in skin renewal and tissue repair [11]. For example, triterpenes such as madecassic acid from *Centella asiatica* stimulate type I collagen synthesis and ECM remodeling in human skin fibroblasts, thereby enhancing extracellular matrix rebuilding and wound repair [12]. In addition, the terpenoid phytohormone, abscisic acid, increases procollagen I and fibronectin production in human dermal fibroblasts, leading to fibroblast-driven tissue regeneration [13]. These diverse dermatological activities make plant extracts valuable anti-aging agents for cosmeceutical and therapeutic applications.

Berries, including blackberry (*Rubus fruticosus*), blueberry (*Vaccinium corymbosum*), cranberry (*Vaccinium macrocarpon*), raspberry (*Rubus idaeus*), strawberry (*Fragaria x ananassa*), and black raspberry (*Rubus coreanus*), are recognized as natural sources for dermatological application because of their high level of bioactive phytochemicals such as flavonoids and polyphenols [14,15]. Among different berries, raspberries and black raspberries contain bioactive phytochemicals, including anthocyanins, ellagic acid, and flavonoids, which exhibit potent antioxidant and anti-inflammatory properties [16]. Previous studies have demonstrated that these berries can mitigate UVB-induced skin damage by reducing oxidative stress and inflammation, thereby offering protective effects against photoaging and skin carcinogenesis. Extracts from these two berry types also maintain skin hydration by regulating gene expression and promoting the formation of the hydrolipidic layer [17,18].

This study provides the first comprehensive investigation of the skin regeneration and hydration properties of *Rubus crataegifolius* Bunge (Rosaceae, RC) fruit extract and provides new insights into its chemical composition and biological activities. Although extracts derived from RC leaves and roots have been examined for various pharmacological effects [19,20], the skin regeneration and hydration properties of its fruit have remained largely unexplored. In this work, we characterized the phytochemical profile of RC fruit extracts obtained through different extraction approaches and putatively annotated their constituents using liquid chromatography–electrospray ionization–time-of-flight mass spectrometry (LC-ESI-TOF-MS). Furthermore, we evaluated their dermatological activities by examining antioxidant capacity and key cellular responses associated with skin regeneration and hydration.

## 2. Results

### 2.1. Phytochemical Profiling of the Extract by High-Resolution LC-ESI-TOF-MS

**Figure 1:**
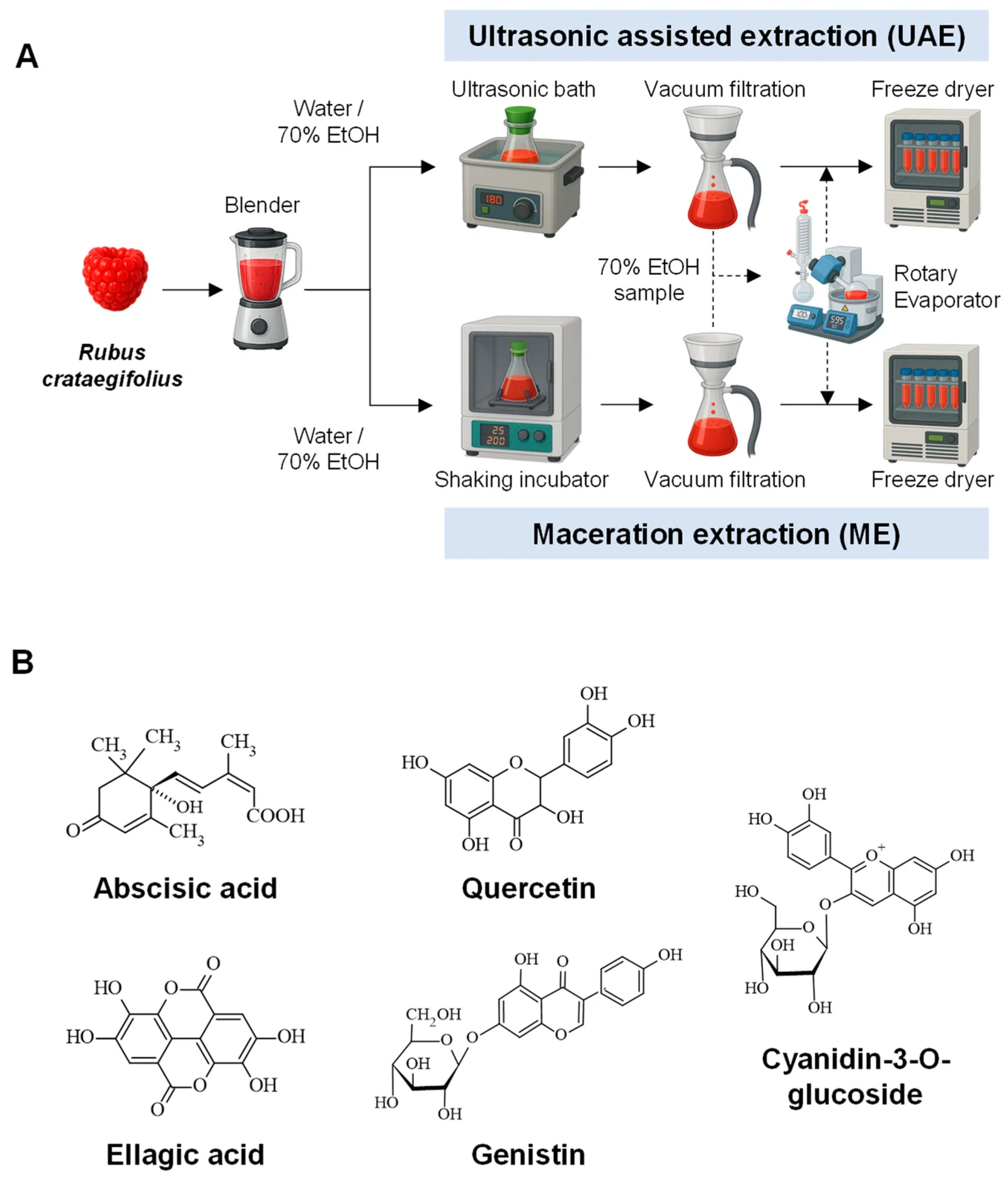
Phytochemical analysis of RC extracts. (**A**) Schematic diagram of extraction procedure. (**B**) Chemical structures of representative compounds, analyzed by LC-ESI-TOF-MS analysis in the RC extracts.

Water and 70% ethanol were selected as two solvents of distinct polarity. Water covers highly polar constituents, and 70% ethanol has also been reported as optimal for polyphenol recovery from fruits [21]. UAE and ME were applied in parallel as representative non-thermal, low-cost methods. This design compared the effect of ultrasonic cavitation with conventional maceration. Various primary and secondary metabolites, including flavonoids, phenolic acids, organic acids, curcumins, tannins, and terpenoids, were putatively annotated in the RC extracts using high-resolution LC-ESI-TOF-MS (Figure 1A,B and Figure S1 and Tables S1–S8). The compounds in the total ion chromatograms (TIC) were putatively annotated based on accurate mass measurement and tandem mass (MS/MS) patterns. Each number in the TIC corresponds to the number in Tables S1–S8, with the corresponding compound shown in bold.

Flavonoids have been reported to be one of the most powerful antioxidants. Many different types of flavonoids were identified in the RC extracts; catechin (Table S1, C_15_H_14_O_6,_ molecular weight 290.079, theoretical mass *m*/*z* 291.0861 [M+H]^+^, observed mass *m*/*z* 291.0860 [M+H]^+^), genistin (Table S1, C_21_H_20_O_10_, molecular weight 432.106, theoretical mass *m*/*z* 433.1125 [M+H]^+^, observed mass *m*/*z* 433.1124 [M+H]^+^), dihydrokaempferol (Table S1, C_15_H_12_O_6,_ molecular weight 288.063, theoretical mass *m*/*z* 289.0706 [M+H]^+^, observed mass *m*/*z* 289.0706 [M+H]^+^), quercetin (Table S1, C_15_H_10_O_7,_ molecular weight 302.043, theoretical mass *m*/*z* 303.0496 [M+H]^+^, observed mass *m*/*z* 303.0496 [M+H]^+^), quercetin glucuronide (Table S1, C_21_H_18_O_13,_ molecular weight 478.075, theoretical mass *m*/*z* 479.0812 [M+H]^+^, observed mass *m*/*z* 479.0813 [M+H]^+^), naringenin (Table S2, C_15_H_12_O_5,_ molecular weight 272.068, theoretical mass *m*/*z* 273.0752 [M+H]^+^, observed mass *m*/*z* 273.0753 [M+H]^+^), naringenin-7-O-glucoside (Table S5, C_21_H_22_O_10_, molecular weight 434.121, theoretical mass *m*/*z* 433.1142 [M−H]^−^, observed mass *m*/*z* 433.1142 [M−H]^−^), hirsutrin (Table S5, C_21_H_20_O_12_, molecular weight 464.095, theoretical mass m/z 463.0883 [M−H]^−^, observed mass *m*/*z* 463.0882 [M−H]^−^), cyanidin-3-O-glucoside (Table S5, C_21_H_20_O_11,_ molecular weight 448.101, theoretical mass *m*/*z* 447.0932 [M−H]^−^, observed mass *m*/*z* 447.0934 [M−H]^−^), and quercetrin (Table S5, C_21_H_20_O_11_, molecular weight 448.101, theoretical mass *m*/*z* 447.0936 [M−H]^−^, observed mass *m*/*z* 447.0935 [M−H]^−^). A major compound, cyanidin-3-O-glucoside, emerged at 10.60–10.72 min in negative ion mode as [M−H]^−^ and [2M−H]^−^ adducts (Tables S5, S6, and S8).

Organic acids, including salicylic acid, protocatechuic acid, indolelactic acid, 3-hydroxy-3-carboxymethyl-adipic acid, and glutaminyl-proline, were putatively annotated. The following secondary metabolites were also putatively annotated: phenolic acid (ellagic acid and gallate), terpenoid (abscisic acid, lucyin A, glabric acid, glabrolide, pyogenic acid, madecassic acid, nimbin and erinacine D), lignan (secoisolariciresinol diglucoside), tannin (3,4-hexahydroxydiphenoylarabinose), and curcumin (artonin C). Interestingly, Ultrasound-Assisted Extraction with distilled water (UAE-DW), Ultrasound-Assisted Extraction with 70% ethanol (UAE-EtOH), Maceration Extraction with distilled water (ME-DW), and Maceration Extraction with 70% ethanol (ME-EtOH) contained abscisic acid eluting at 23.13–23.15 min. In positive ion mode, two types of adducts ([M+H−H_2_O]^+^ and [M+Na]^+^) were putatively annotated (Table S1: C_15_H_20_O_4,_ molecular weight 264.136, theoretical mass *m*/*z* 247.1322 [M+H−H_2_O]^+^, observed mass *m*/*z* 247.1322 [M+H−H_2_O]^+^, theoretical mass *m*/*z* 287.1251 [M+Na]^+^, observed mass *m*/*z* 287.1250 [M+Na]^+^). Meanwhile, the adduct [M−H]^−^ of abscisic acid was observed in negative ion mode (Table S5: C_15_H_20_O_4,_ molecular weight 264.136, theoretical mass *m*/*z* 263.1294 [M−H]^−^, observed mass *m*/*z* 263.1295 [M−H]^−^).

### 2.2. Flavonoid Content and Antioxidant Activity

**Figure 2:**
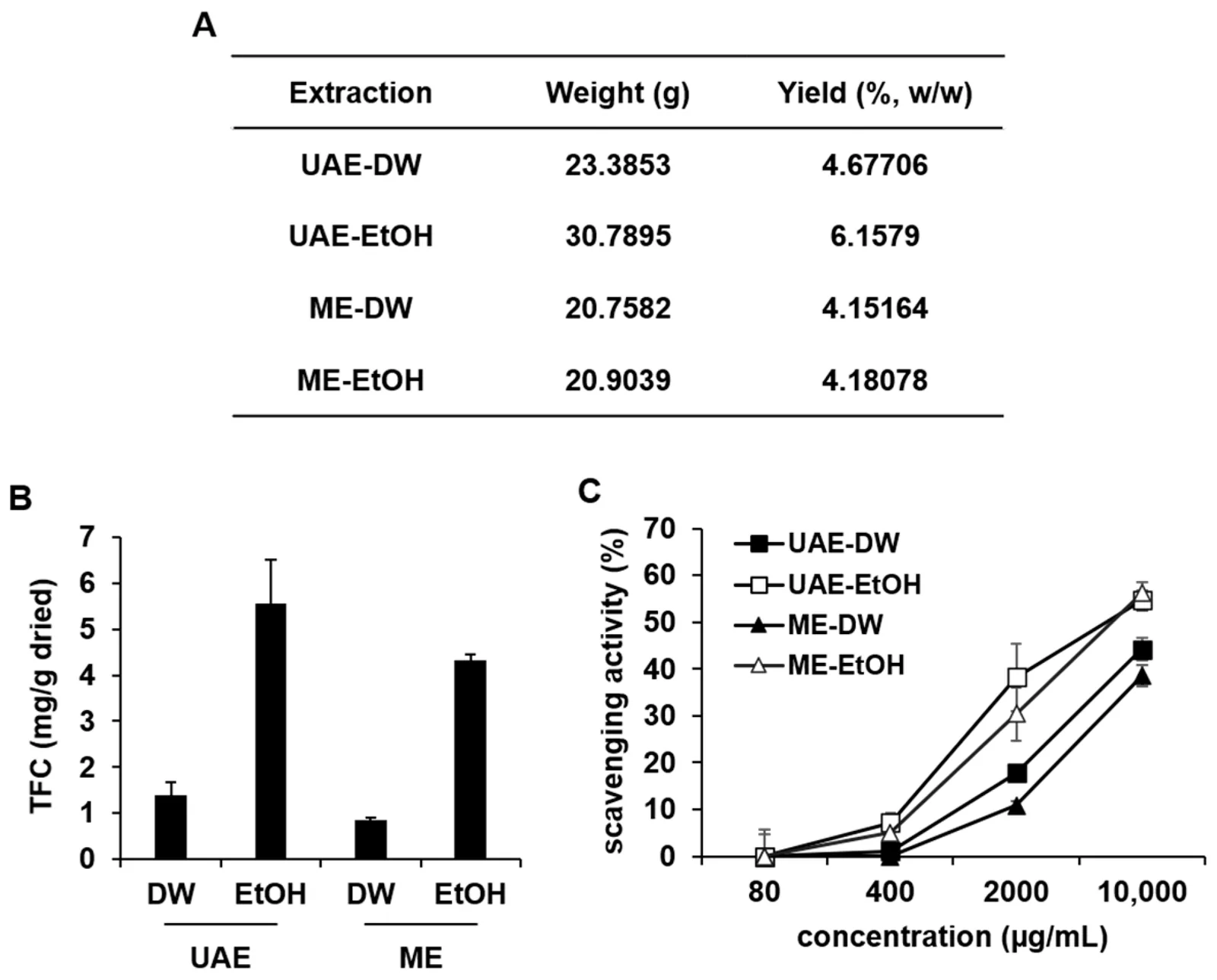
Total flavonoid content (TFC) and antioxidant activity of RC extracts. (**A**) Extraction yield of RC extracts obtained from four different methods. The yield was calculated based on the weight of freeze-dried extracts relative to the initial raw material weight. (**B**) Total flavonoid content (TFC) of RC extracts. (**C**) In vitro antioxidant activity of RC extracts using DPPH assay. All data represent the mean value ± standard deviation of three independent experiments.

The yield of RC extracts ranged from 4% to 6%, with no significant differences observed between extraction methods (Figure 2A). Since phytochemicals, including flavonoids and glycosides, were detected in RC extracts (Figure 1B), the total flavonoid content of RC extracts was determined using the aluminum chloride colorimetric method. Compared to RC extracts obtained using DW as a solvent, those extracted with ethanol exhibited higher total flavonoid content (Figure 2B). These results are consistent with previous reports, which show that ethanol-based extraction yields higher flavonoid content [22]. Since extracts with high flavonoid content exhibit antioxidant activity [23], antioxidant activity was also assessed using the 2,2-diphenyl-1-picrylhydrazyl (DPPH) radical scavenging method. In general, extracts obtained using EtOH as a solvent exhibited higher DPPH radical scavenging activity compared to water-based extracts. The DPPH radical scavenging activity ranged from 7.18 ± 2.09% to 54.63 ± 2.05% for UAE-EtOH and from 5.00 ± 3.33% to 56.23 ± 2.41% for ME-EtOH, whereas water-based extracts showed a range of 1.09 ± 1.91% to 44.22 ± 2.42% for UAE-DW and 10.85 ± 0.98 to 38.54 ± 2.20% for ME-DW, with activity increasing in a concentration-dependent manner (Figure 2C). These results demonstrate that RC fruit extracts have a high flavonoid content and exhibit flavonoid-based antioxidant activity.

### 2.3. Fibroblast-Activating Effects of RC Extracts

**Figure 3:**
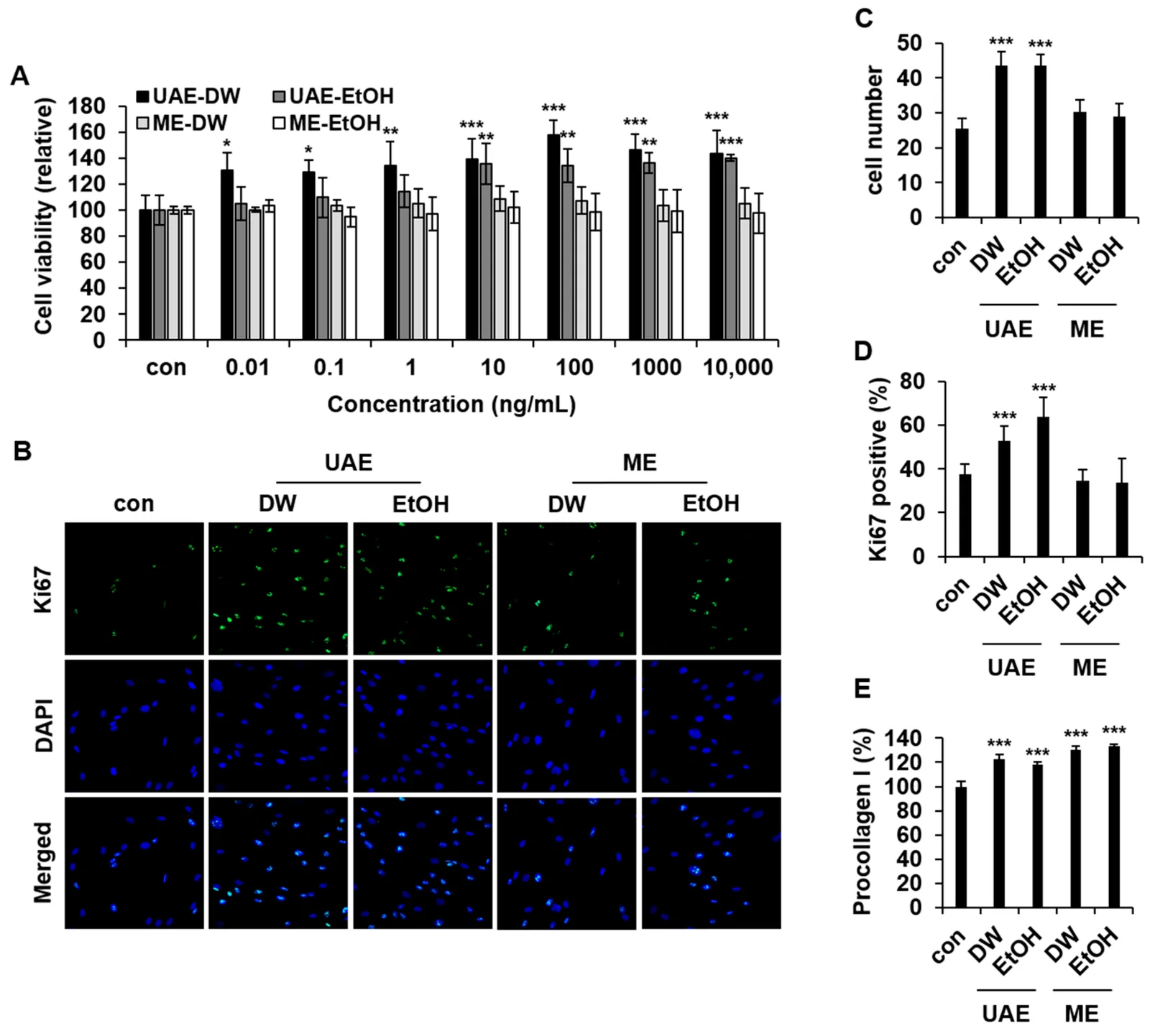
Fibroblast-activating effects of RC extracts. (**A**) Proliferation-inducing effects of RC extracts in dermal fibroblasts. Hs68 cells (3 × 10^3^/well) were seeded and then treated with different concentrations (0.01–10,000 ng/mL) of RC extracts. After 48 h of incubation, cell viability was determined by a CCK-8 assay. (**B**) Immunofluorescence images of Ki-67-stained Hs68 fibroblasts. Ki-67 (green) and nuclear immunostaining on Hs68 cells were performed with the SolA15 clone and DAPI (blue). (**C**) Total number of DAPI-positive cells. (**D**) The quantification graph of Ki-67-positive cells in Hs68 after 48 h incubation at 1000 ng/mL of RC extracts (*n* = 7). (**E**) Collagen-inducing effect of extracts. After 48 h of incubation with extracts, cultured media from Hs68 cells were harvested to assess pro-collagen type I levels. Pro-collagen type I was measured by ELISA. Error bars represent the mean ± SD from the average of triplicate experiments. *** *p* < 0.001, ** *p* < 0.01. * *p* < 0.05.

Since plant-derived extracts, having antioxidant flavonoids, are effective in protecting skin cells from stress and aging [8,11], various anti-aging activities of RC extracts, including fibroblast proliferation and collagen synthesis, were examined. To assess the proliferation-inducing effect of RC extracts, Hs68, a human foreskin dermal fibroblast line, was treated with RC extracts at concentrations from 0.01 to 10,000 ng/mL. After 48 h of incubation, cell proliferation was determined using CCK-8 solution. Fibroblast proliferation was induced by UAE-DW and UAE-EtOH in a dose-dependent manner (Figure 3A). In the group treated with UAE-DW, cell proliferation was induced at concentrations ranging from 10 ng/mL (139.22 ± 15.25%) to 10,000 ng/mL (143.21 ± 18.04%), with the highest proliferation observed at 100 ng/mL (157.5 ± 11.55%). The group treated with UAE-EtOH also showed proliferative activity at concentrations from 10 ng/mL (135.41 ± 15.63%) to 10,000 ng/mL (140.01 ± 2.46%). Interestingly, the other extracts, including ME-DW and ME-EtOH, did not induce fibroblast proliferation. To validate the proliferation-inducing effect of RC extracts in fibroblasts, Ki-67, a prominent proliferation marker [24,25], was also detected by immunofluorescence staining (Figure 3B). In Hs68 cells treated with 1000 ng/mL of RC extracts, the average number of DAPI-stained cells significantly increased to 44 cells per square in both the UAE-DW and UAE-EtOH treatments, compared to 26 cells per square in the control (Figure 3C). In addition, the percentage of Ki-67 positive cells increased to 53% and 64% in UAE-DW and UAE-EtOH treatments, respectively, compared to 37% in the control (Figure 3D). Ki-67 upregulation confirms that cell proliferation is actively promoted by RC extracts. 

Among the various fibroblast functions related to skin homeostasis and anti-aging, collagen synthesis is one of the most important properties [26]. Since RC extracts induced fibroblast proliferation, collagen synthesis was subsequently assessed in the culture medium of RC extract-treated fibroblasts. Hs68 cells were incubated with RC extracts at a concentration of 1 μg/mL. After 48 h of incubation, collagen concentration in cultured medium was determined using COL1A1 ELISA. All types of RC extracts enhanced collagen concentration in the culture medium (Figure 3E). The greatest increase in collagen concentration of 133.59 ± 1.45%, compared to the control, was observed with ME-EtOH treatment. Although RC extracts prepared using EtOH solvent showed antioxidant activity, fibroblast proliferation was specifically induced by UAE-derived extracts, while collagen synthesis was enhanced across all four extracts regardless of solvent. Based on these results, the fibroblast-activating effects of RC extracts are unrelated to their antioxidant activity.

### 2.4. Wound-Healing Activity of RC Extracts

**Figure 4:**
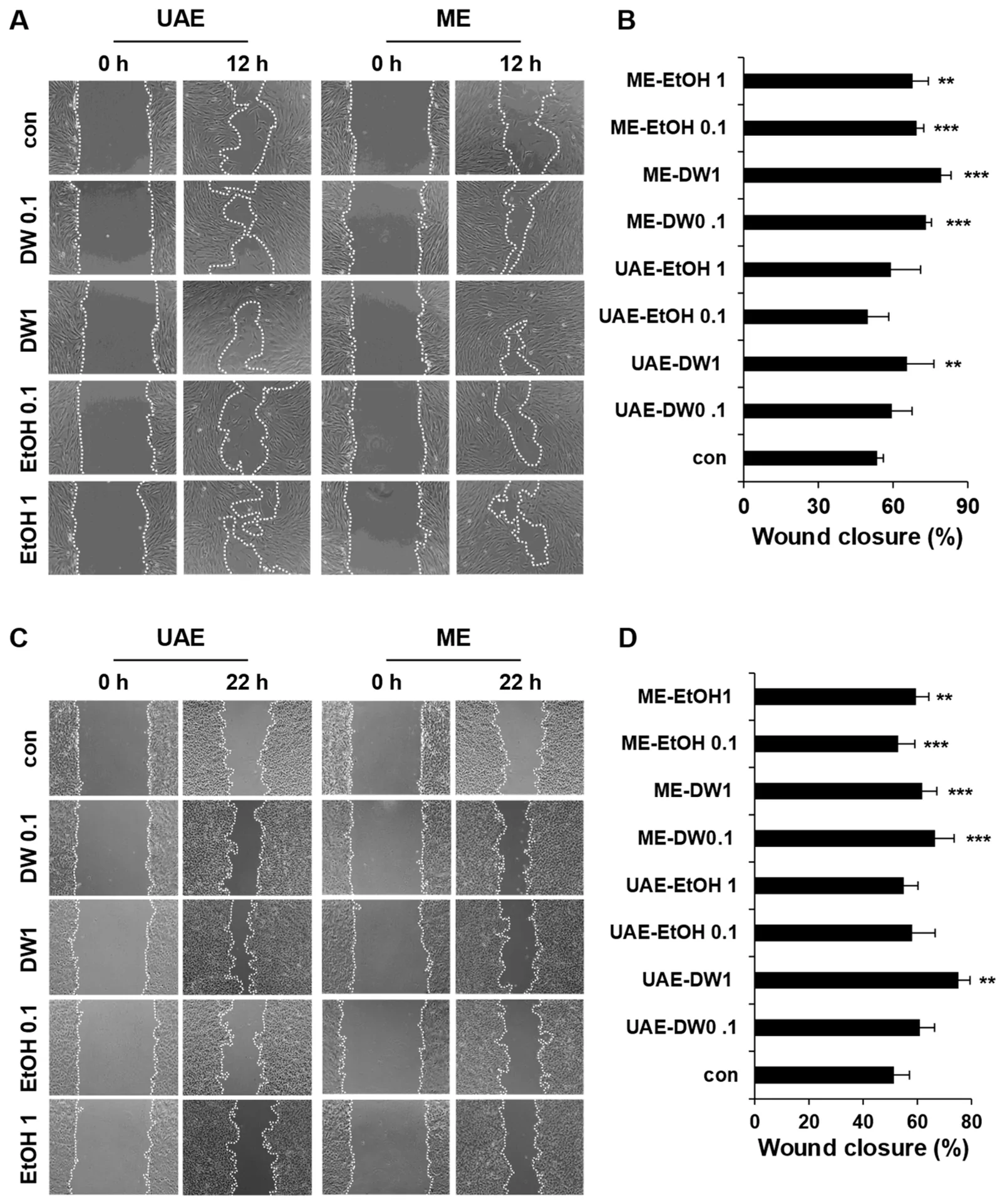
Wound-healing effects of RC extracts. Wound-healing effect of RC extracts in Hs68 and HaCaT cells. The cell layer was scratched with a micropipette tip to make a wound and then incubated with RC extracts for 12 and 22 h, respectively. Photos were taken after treatment with or without RC extracts (0.1, 1 µg/mL). Cells were outlined with dotted lines. (**A**) Representative images of scratched wounds in Hs68 cells. (**B**) Graph illustrating relative wound closure area after RC extract treatment in Hs68 cells. (**C**) Representative images of scratched wounds in HaCaT cells. (**D**) Graph illustrating relative wound closure area after RC extract treatment in HaCaT cells. Error bars represent the mean ± SD from the average of triplicate experiments. *** *p* < 0.001, ** *p* < 0.01.

Since RC extracts exhibited fibroblast-activating activity, the in vitro wound-healing effect of RC extracts was also evaluated using a scratch wound-healing assay. After making a scratch, Hs68 and HaCaT human keratinocyte cells were treated with RC extracts for 12 and 22 h, respectively. Cell migration into the scratch wound area was monitored under a microscope. To clearly outline the migrated cells, they were marked with a dotted line. ME-DW and ME-EtOH extracts significantly increased the wound-closing area in Hs68 cells (Figure 4A). Compared to the control, 53.57 ± 2.52%, ME-DW at 0.1 μg/mL and 1 μg/mL induced relative wound closure of 73.17 ± 2.25% and 79.33 ± 3.94%, respectively, while ME-EtOH at 0.1 μg/mL and 1 μg/mL induced relative wound closure of 69.49 ± 2.82% and 67.84 ± 6.30%, respectively (Figure 4B). ME extracts only exhibited significant wound-healing activity in fibroblasts. Scratched wound closure of HaCaT was induced by UAE-DW and ME-DW extracts (Figure 4C). Compared to the control, 51.39 ± 5.72%, UAE-DW at 0.1 μg/mL and 1 μg/mL induced relative wound closure of 61.00 ± 5.37% and 75.24 ± 4.21%, respectively, while ME-DW at 0.1 μg/mL and 1 μg/mL induced closure rates of 66.61 ± 6.97% and 61.80 ± 5.40%, respectively (Figure 4D). Extracts using EtOH did not have a significant wound-healing effect in HaCaT cells.

### 2.5. Safety of RC Extracts

**Figure 5:**
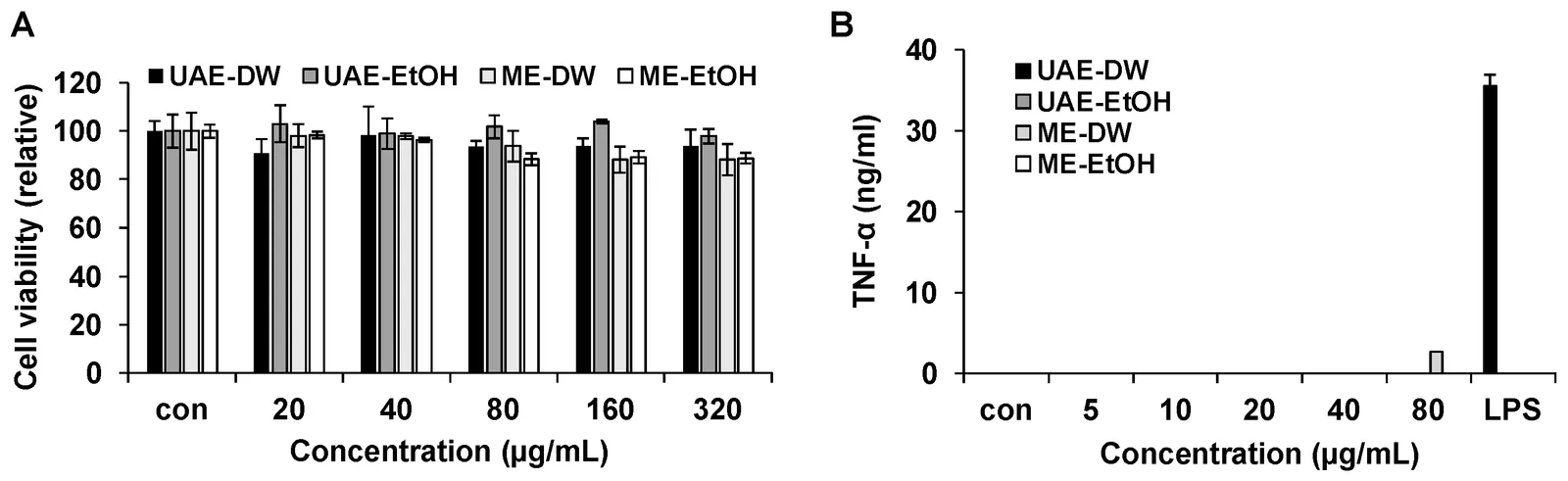
Non-cytotoxicity and anti-inflammatory activity of RC extracts. (**A**) Cytotoxicity of RC extracts. Vero cells were used to test the cytotoxicity of RC extracts, ranging from 20 to 320 µg/mL. (**B**) Pro-inflammatory activity of RC extracts in macrophages. RAW 264.7 cells were treated with a high concentration of RC extracts. LPS-treated cells (5 ng/mL) were used as a positive control; buffer-treated cells were used as a negative control. To test pro-inflammatory potential, TNF-α was determined by ELISA. Error bars represent the mean ± SD from the average of triplicate experiments.

To assess the cytotoxicity of RC extracts, Vero cells, a monkey kidney epithelial cell line, were used. Vero cells were treated with RC extracts at concentrations from 0 to 320 μg/mL, a range substantially exceeding the biologically active concentrations used in the preceding assays. After 24 h of treatment, cell viability was determined. Although 10% cytotoxicity was observed at concentrations higher than 80 μg/mL, RC extracts exhibited non-cytotoxicity at concentrations at least 80-fold higher than the effective concentration (Figure 5A).

Inflammation is a major adverse effect in dermatological applications. Although flavonoids detected in RC extracts possess anti-inflammatory activity [27], certain phytochemicals may induce an inflammatory response. To assess the pro-inflammatory potential of RC extracts, the levels of the pro-inflammatory cytokine, TNF-α, were measured after RC extract treatment in macrophages. RAW 264.7 cells, a mouse macrophage cell line, were incubated with RC extracts at concentrations from 0 to 80 μg/mL. Lipopolysaccharide (LPS) was used as a positive control. After 6 h, TNF-α ELISA was performed to assess the pro-inflammatory potential of RC extracts. Except for ME-DW at a concentration of 80 μg/mL, all RC extracts induced an immunogenic response in macrophages (Figure 5B). These results showed that four different RC fruit extracts are non-cytotoxic and non-immunogenic.

### 2.6. Clinical Efficacy of RC Extracts on Skin Hydration

**Figure 6:**
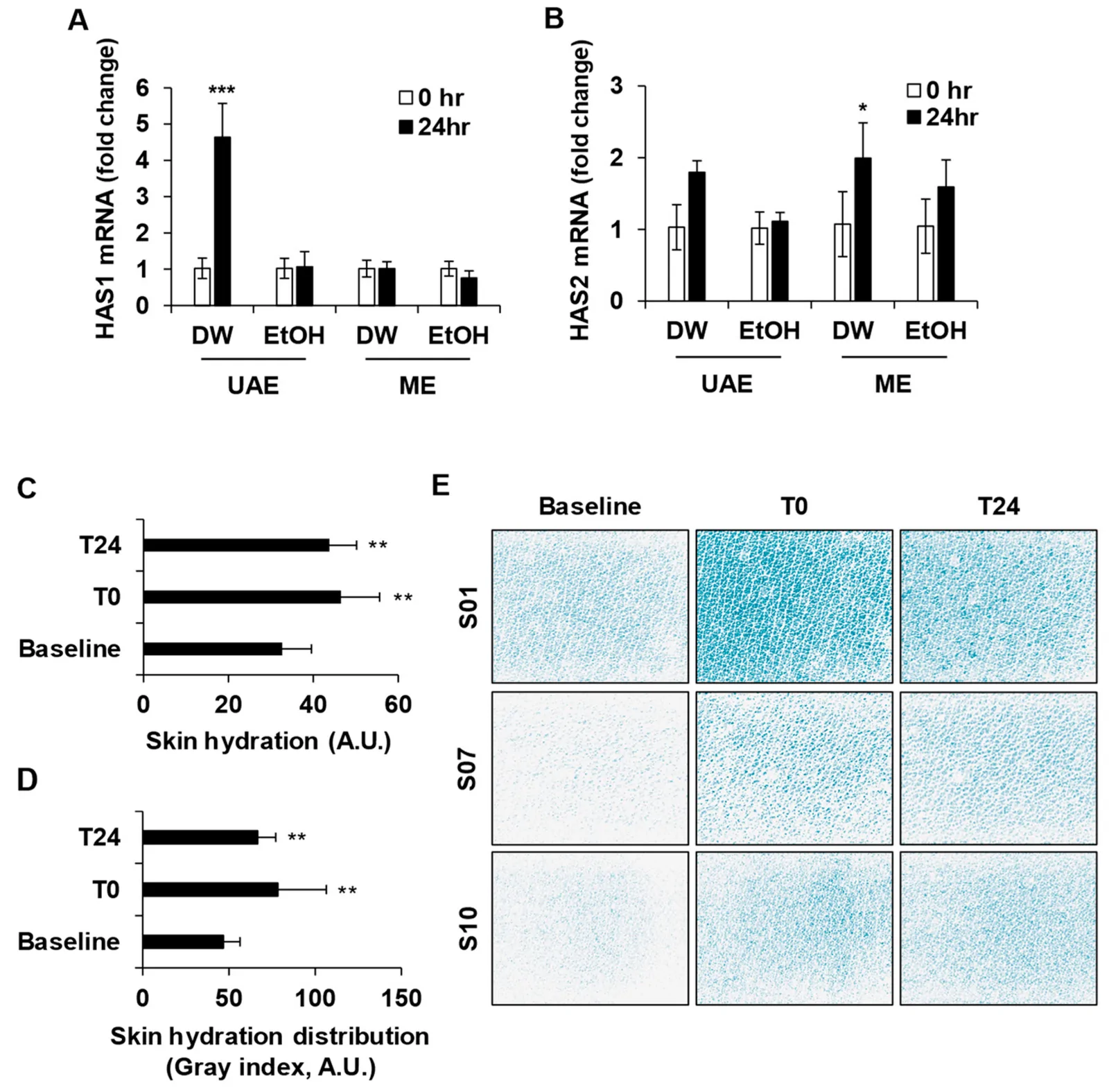
Hydration efficacy of RC extracts on skin. Hydration effect of RC extracts in keratinocytes (**A**,**B**). Total mRNA was extracted after 24 h treatment with 20 μg/mL RC extracts. The expression levels of *HAS1* (**A**) and *HAS2* (**B**) were quantified by qRT-PCR. Relative gene expression of HAS1 and HAS2 was normalized to GAPDH. Clinical evaluation of skin hydration with UAE-DW extract in 12 participants (**C**–**E**). Twelve participants applied 20 μg/mL UAE-DW for 24 h. (**C**) Skin hydration was assessed using the Corneometer^®^ CM 825 device (Courage Khazaka Electronic GmbH, Cologne, Germany) at baseline, immediately after application, and 24 h post-application. (**D**) Skin hydration distribution was evaluated using the MoistureMap MM200 device (Courage Khazaka Electronic GmbH, Cologne, Germany) at baseline, immediately after application, and 24 h post-application. (**E**) Hydration images of volar forearm skin in three of the volunteers were obtained using the MoistureMap MM200 device. *** *p* < 0.001, ** *p* < 0.01, * *p* < 0.05.

The relative mRNA expression levels of *HAS1* and *HAS2* in human keratinocytes were quantified using qRT-PCR. Only UAE-DW treatment induced the expression of both *HAS1* and *HAS2* mRNA (Figure 6A,B). Since *HAS1* and *HAS2* are associated with skin hydration, the clinical efficacy of UAE-DW was subsequently evaluated.

Twelve healthy female volunteers were enrolled in the clinical study. The mean age of the participants was 46.42 ± 6.30 years (range: 38–59 years). No participant discontinued due to noncompliance. Demographic data of participants were collected (Table S9). Twelve participants applied UAE-DW for 24 h. Skin moisture content was significantly increased immediately after application (T0) and remained elevated at 24 h post-application (T24) compared with baseline, with mean percentage changes of 45.40% and 36.44%, respectively (Figure 6C). Skin hydration distribution was also significantly increased by UAE-DW at both time points, with mean percentage changes of 69.68% and 44.21%, respectively (Figure 6D). Representative hydration distribution images from participants S01, S07, and S10 demonstrated that UAE-DW application enhanced skin hydration distribution (Figure 6E). No adverse events were observed during the study period, and none of the participants had medical conditions or medication histories that could influence the outcomes. This clinical study was limited to 12 female participants over a 24 h period and did not include a vehicle-treated or untreated control site; therefore, the observed increase in skin hydration cannot be attributed solely to the *Rubus crataegifolius* extract or interpreted as a long-term benefit. Nevertheless, the results are consistent with the in vitro dermatological activity of the extract demonstrated in human fibroblasts and keratinocytes.

## 3. Discussion

This study demonstrates that RC fruit extracts exert diverse dermatological activities, including fibroblast proliferation, collagen synthesis, wound healing, and skin hydration. The observed activities varied according to extraction method and solvent: ultrasound-assisted extracts most strongly enhanced fibroblast proliferation, maceration extracts were more effective in promoting wound healing, and the ultrasound-assisted water extract selectively induced *HAS1* expression. These extract-specific patterns may arise, at least in part, from differences in phytochemical composition produced by each solvent and extraction method, which, in turn, influence the skin cell functions promoted by each extract.

Beyond these differential effects, RC fruit extracts also stimulated collagen synthesis in fibroblasts and promoted keratinocyte proliferation, indicating a coordinated action on both dermal and epidermal compartments. The concurrent upregulation of hyaluronic acid synthase genes (*HAS1* and *HAS2*) further supports an active role in extracellular matrix remodeling and skin hydration. Collectively, these findings suggest that RC fruit extracts do not merely protect skin cells against oxidative stress but actively drive regenerative processes—including cell proliferation, matrix production, and wound closure—under non-stressed conditions. The distinct activity profiles of the individual extracts imply that specific phytochemical subsets enriched by each extraction condition may preferentially engage different cellular pathways involved in skin regeneration and barrier function.

These regenerative properties are particularly relevant to anti-aging research, which has grown in importance as human lifespan has extended. Anti-aging agents aim to reduce wrinkles and pigmentation and restore skin homeostasis through cosmeceutical ingredients such as retinoids, quercetin, polyphenols, and vitamins C and E [28]. These cosmeceutical ingredients share common effects, including stimulation of epidermal turnover, enhancement of collagen synthesis, antioxidant activity, and reduction of hyperpigmentation; however, these can sometimes cause unexpected irritation and inflammation of the skin [29]. For example, retinoids commonly cause erythema, dryness, peeling, burning sensation, and increased photosensitivity, with concerns about chemical instability and potential teratogenicity [30]. Consequently, there is growing interest in identifying mild, non-irritating natural ingredients, including dermatologically effective phytochemicals and phytohormones, as alternatives for anti-aging applications.

Within this context, we focused on *Rubus crataegifolius* (RC), a species within the genus *Rubus*, due to scientific evidence supporting the dermatological potential of related berries such as *Rubus idaeus* (raspberry) and *Rubus coreanus* (black raspberry) [31,32]. Whereas previous dermatological studies on *Rubus* and other berry extracts have focused largely on protective, redox-driven mechanisms, our findings reveal a distinct axis centered on the active induction of skin regeneration and hydration. For example, ethanol extracts of *Rubus coreanus* and *Rubus idaeus* counteract UVB-induced photoaging by suppressing matrix metalloproteinase production and restoring procollagen in dermal fibroblasts [33,34]. A water extract of *R. coreanus* exerts antioxidant and anti-inflammatory effects in a keratinocyte model of atopic dermatitis [35]. In contrast, RC fruit extracts in this study promoted dermatological activity under normal, non-stressed conditions.

To identify the constituents underlying these activities, RC fruit extracts were analyzed by LC-ESI-TOF-MS. A diverse array of phytochemicals was putatively annotated, including flavonoids, phenolic acids, organic acids, and terpenoids. Although ethanol-based extracts contained higher total flavonoid content and stronger antioxidant capacity than water-based extracts, dermatological activities did not consistently parallel flavonoid content or antioxidant activity.

Therefore, these activities may be related, at least in part, to terpenoids, which were detected in all types of RC extracts. In LC-MS/MS analysis, abscisic acid (ABA) was consistently detected in four different RC extracts. ABA has been reported to promote procollagen synthesis and fibroblast activation [13], and to modulate inflammatory responses in keratinocytes [36]. Along with ABA, several terpenoids—including lucyin A, glabric acid, glabrolide, pyogenic acid, madecassic acid, nimbin, and erinacine D—were also putatively annotated. Many of these phytochemicals have been reported to exhibit dermatological effects such as wound repair, anti-inflammatory responses, or extracellular matrix regulation [37,38]. These findings suggest that the biological activities of RC fruit extracts may involve the combined contribution of multiple annotated compounds.

Overall, the extract-specific activity profiles underscore the role of extraction conditions in determining the phytochemical composition of RC fruit extracts and, consequently, their dermatological functionality, supporting the potential of RC fruit as a natural functional ingredient for cosmeceutical and skin care applications.

## 4. Materials and Methods

### 4.1. Material Preparation

The fruits of *Rubus crataegifolius* (RC) were collected in Gimhae, Republic of Korea, in 2023. The botanical identification was made by Chang-Woo Hyun (National Institute of Biological Resources, Incheon, Republic of Korea), and a voucher specimen was deposited at the NIBR Herbarium (No. NIBRVP0000884561). RC fruits were stored at −80 °C. Before use, frozen fruits were thawed at room temperature. After thawing, RC fruits were washed with distilled water (DW) to remove dust and surface debris.

### 4.2. Extraction

For each extraction, five hundred grams of fresh RC fruit were milled to obtain a homogenized sample. Distilled water (DW) or 70% ethanol was then added to the homogenized sample at a 1:1 (*v*/*v*) sample-to-solvent ratio. For maceration extraction (ME), the mixture was incubated with shaking at 200 rpm for 24 h at 25 °C. Ultrasound-Assisted Extraction (UAE) was performed using an ultrasonic water bath (DAIHAN Scientific, Wonju, Republic of Korea) for 1 h. After extraction, the mixtures were filtered under vacuum using Whatman filter paper (Whatman, Maidstone, UK). The filtrates were then concentrated using a rotary evaporator (Rotavapor R-3, BUCHI, Flawil, Switzerland) and subsequently freeze-dried (IlShin Bio Base, Yangju, Republic of Korea) for 5 days. The dried extracts were stored at −80 °C until use. The extraction yield (expressed in %) was calculated as shown in the equation:

$$Extraction\;yield\left(\%\right)=\frac{dried\;extract\left(g\right)}{initial\;RC\;fruits(g)}\times 100$$

### 4.3. Phytochemical Profiling of the Extract by High-Resolution LC-ESI-TOF-MS

Liquid chromatography was performed using an Ultimate 3000 (Thermo Scientific, Waltham, MA, USA). Chromatographic separation was conducted at 45 °C on a Waters Cortecs C18 column (2.1 mm diameter × 150 mm length, 1.6 μm particle size) with a flow rate of 0.25 mL/min. A gradient elution was carried out using two mobile phases: mobile phase A was water containing 0.1% formic acid, and mobile phase B was acetonitrile containing 0.1% formic acid. The gradient elution was as follows: the mobile phase was gradually eluted from 97% mobile phase A and 3% mobile phase B to 0% mobile phase A and 100% mobile phase B over 50 min.

A Triple TOF 5600+ instrument (AB SCIEX, Framingham, MA, USA) was used for TOF-MS analysis with electrospray ionization (ESI) (AB SCIEX, Framingham, MA, USA) [39]. Full mass scan and MS/MS scan ranges were set at *m*/*z* 100–2000 and *m*/*z* 30–2000, respectively. The pressures for both ion source 1 (nebulizing gas) and ion source 2 (heating gas) were set at 50 psi. The desolvation temperature was 500 °C, with curtain gas pressure at 25 psi. The ion spray voltage floating was set at 5.5 kV for positive ionization mode and at 4.5 kV for negative ionization mode. For MS/MS, nitrogen was used as the collision gas with a collision energy of 35 ± 15 eV and −35 ± 15 eV for positive and negative ionization modes, respectively.

Compound annotation was performed based on high-resolution accurate-mass measurements and MS/MS fragmentation patterns. MS/MS spectral library searching was performed using the CES Confirmation Library Search workflow with all available libraries selected. The library-search mass tolerance was set to 0.4, with a purity threshold of 0.05. Candidate metabolites were annotated based on agreement between the observed and theoretical *m*/*z* values, the assigned ion/adduct form, and consistency of the MS/MS fragmentation pattern with library-search results. The individual mass errors (ppm) are provided in Tables S1–S8. Because authentic reference standards were not used for confirmation of retention time and MS/MS spectra, the compounds are reported as putatively annotated metabolites rather than unequivocally identified metabolites.

### 4.4. Total Antioxidant Activity Assay Using the DPPH Method

The 2,2-diphenyl-1-picrylhydrazyl (DPPH) radical-scavenging activity was performed to evaluate antioxidant activities of RC extracts, using the DPPH antioxidant assay kit (Dojindo, Kumamoto, Japan). Following the manufacturer’s instructions, the DPPH radical scavenging activity of different concentration extracts was quantified. Briefly, 20 µL of sample/standard was added to 100 µL of DPPH working solution. The mixture was left in the dark at room temperature for 30 min. The absorbance was measured at 517 nm using a microplate reader (BioTek Synergy, Winooski, VT, USA). The percentage of DPPH radical scavenging capacity is expressed as shown in the following equation:

$$\%\;radical\;scavenging\;capacity=\frac{A_{b}-A_{s}}{A_{b}}\times 100$$

where *A_b_* is the absorbance of the blank and *A_s_* is the absorbance of each sample.

### 4.5. Total Flavonoid Content (TFC) Assay

An aluminum chloride colorimetric method with a plant flavonoid assay kit (Novus Biologicals, Centennial, OH, USA) was used to determine the total flavonoid content (TFC) of RC extracts. After incubating RC extracts with aluminum reagent for 30 min, the absorbance was measured at 510 nm using NanoDrop2000 (Thermo Scientific). Total flavonoid content of RC extracts was calculated using the quercetin standard curve and converted to quercetin equivalents (QEs/mg of RC extracts).

### 4.6. Cell Proliferation Assay

HaCaT (ATCC, PCS-200-011, Manassas, VA, USA) and Hs68 (ATCC, CRL-1635) cells were cultured in Dulbecco’s Modified Eagle Medium (DMEM; HyClone, Logan, UT, USA) supplemented with 10% (*v*/*v*) fetal bovine serum (FBS; HyClone), 1% (*v*/*v*) penicillin/streptomycin (HyClone). Cells were maintained at 37 °C in a humidified atmosphere containing 5% CO_2_. Hs68 and HaCaT (3 × 10^3^/well) cells were seeded into a 96-well plate (SPL, Pocheon, Republic of Korea). Cells were then treated with RC extracts at concentrations ranging from 0 to 10,000 ng/mL. After 48 h of incubation, cell proliferation was measured using Cell Counting Kit-8 (CCK-8, Dojindo) following the manufacturer’s instructions. Briefly, 10 μL of CCK-8 solution was added to cultured media and incubated in a CO_2_ incubator for 90 min. The absorbance was measured at 450 nm using a microplate reader (BioTek Synergy).

### 4.7. Immunofluorescence Staining

For immunofluorescence staining, Hs68 cells were washed three times with PBS and then fixed using 4% paraformaldehyde (PFA) for 15 min. Cells were permeabilized with 0.3% Triton X-100 for 15 min and blocked with 2% (*w*/*v*) bovine serum albumin (BSA) in PBS for 1 h at RT. Cells were then stained with anti-Ki-67 antibody conjugated with Alexa Fluor 488 (SolA15 clone, Invitrogen, Waltham, MA, USA) for 2 h. For nuclear counterstaining, cells were stained with 4′,6-diamidino-2-phenylindole (DAPI, 1:1000, #D1306, Invitrogen) for 10 min. After staining, cells were mounted on glass slides. Imaging was performed using confocal microscopy (LSM800, Zeiss, Oberkochen, Germany) with laser wavelengths of 405 nm and 488 nm. Analysis was performed using ZEN Blue 2.3 Software (Carl Zeiss).

### 4.8. Scratch Wound Migration Assay

For the scratch wound migration assay, Hs68 (8.75 × 10^4^/well) and HaCaT (1.8 × 10^5^/well) cells were seeded in a 24-well plate (SPL). Before scratching the cells, the medium was replaced with serum-free medium. After 2 h of incubation, cells were scratched using a pipette tip. To remove debris, scratched cells were washed with serum-free medium and treated with RC extracts at concentrations ranging from 0 to 1000 ng/mL. Images of wound closure were captured with an inverted light microscope (Nikon, Tokyo, Japan) equipped with a digital camera (ToupTek, Hangzhou, China) at each time point. The ImageJ software 1.54p (NIH, Bethesda, MD, USA) was used to measure the area of the scratch.

### 4.9. Enzyme-Linked Immunosorbent Assay (ELISA)

A human pro-collagen I α1 kit (R&D Systems, Minneapolis, MN, USA) was used to monitor the secretion of collagen type I in RC extract-treated Hs68 cells. Hs68 cells were treated with RC extracts (1000 ng/mL) for 48 h. The culture medium was then harvested and centrifuged at 1000 rpm to remove cells. The supernatant was used for ELISA. The absorbance was measured at 450 nm using a microplate reader (BioTek Synergy). To determine TNF-α levels in RAW 264.7 cell (ATCC, TIB-71) culture medium, a mouse TNF-α ELISA kit (R&D Systems) was used according to the manufacturer’s instructions. RAW 264.7 cells were treated with RC extracts at various concentrations (5, 10, 20, 40, and 80 μg/mL) for 6 h.

### 4.10. Quantitative Real-Time PCR (qRT-PCR)

The transcript levels of *HAS1* and *HAS2* were measured using quantitative real-time PCR (qRT-PCR). HaCaT (2 × 10^5^/well) cells were seeded in 6-well plates and treated with RC extracts. After 24 h of incubation, total RNA was extracted using AccuPrep^®^ Universal RNA Extraction Kit (Bioneer, Daejeon, Republic of Korea). The cDNA was synthesized from 100 ng total RNA using NICSROgene™ Reverse Transcription Kit (Bionics, Seoul, Republic of Korea). qPCR amplification of selected genes was carried out using a 7500 Real-Time PCR System (Applied Biosystems, Waltham, MA, USA) and SYBR qPCR master mix (Epigen, Cheongju, Republic of Korea). GAPDH was used as a reference gene. The relative expressions of *HAS1* and *HAS2* were calculated using the delta-delta Ct method.

### 4.11. Clinical Study of Hydration

A human application test was conducted to evaluate the short-term moisturization effects of RC fruit extract. The study was performed at the AllLive Clinical Trial Research Center (Gyeonggi-do, Republic of Korea), where all participants were recruited. Twelve healthy female volunteers (38–59 years) meeting the eligibility criteria, including healthy adults with no acute or chronic dermatological conditions on the forearm, no recent (≤1 month) use of topical corticosteroids or functional cosmetics, no pregnancy or suspicion thereof, and no history of hypersensitivity to cosmetic ingredients, were enrolled in the study, while individuals with active skin lesions, photosensitivity, recent dermatologic procedures, or relevant systemic diseases were excluded. All assessments were conducted under controlled environmental conditions (22 ± 2 °C, 50 ± 5% RH) with no airflow, no direct sunlight, and constant illumination, following a 20 min stabilization period. The formulation contained 20 µg/mL RC extract (UAE-DW) in sterile distilled water with 1% 1,2-hexanediol. A 0.5 mL volume of the formulation was applied to a 3 × 4 cm area on the forearm of each of the participants for 24 h. Skin hydration was measured using Corneometer^®^ CM 825 (Courage Khazaka Electronic GmbH, Cologne, Germany). The hydration distribution was assessed using a MoistureMap MM200 (Courage Khazaka Electronic GmbH). Measurements were obtained at baseline, immediately post-application, and 24 h post-application, with each value representing the mean of three repeated readings. Participants were instructed to report any irritation or discomfort, and the application sites were inspected for adverse reactions throughout the evaluation period. No abnormal responses were observed during the study. This clinical assessment was conducted in accordance with institutional guidelines for cosmetic human use testing, and the study protocol was approved by the Institutional Review Board (IRB) of the AllLive bioethics committee (IRB number: IRB-E2401-002). This study was classified as a cosmetic human application test under Korean Ministry of Food and Drug Safety (MFDS) guidelines and was therefore not subject to prospective registration as a clinical trial. This study was conducted in accordance with the Declaration of Helsinki and adhered to all ethical standards for the protection of participant rights and welfare. All participants provided written informed consent after being fully informed of the study purpose, procedures, potential benefits, risks, confidentiality protections, and their rights to voluntary participation and withdrawal.

### 4.12. Statistical Analysis

Statistical analysis was performed using GraphPad Prism 9.1.1 (GraphPad, San Diego, CA, USA). Data involving two factors were analyzed by two-way ANOVA, while data involving a single factor were analyzed by one-way ANOVA. Dunnett’s multiple-comparison test was used for comparisons against the control. *p* < 0.05 was considered to indicate a statistically significant difference. Results were presented as mean ± standard deviation (SD).

Statistical analysis in the clinical study was performed on the per-protocol (PP) set using SPSS Statistics 27 Standard (IBM, Armonk, NY, USA). Normality was assessed using the Shapiro–Wilk test, with a *p*-value ≥ 0.05 indicating a normal distribution. Pre-and post-application changes at each time point were compared using a paired *t*-test for normally distributed data and the Wilcoxon signed-rank test for non-normally distributed data. *p* < 0.05 was considered to indicate a statistically significant difference.

## Data Availability

The original contributions presented in this study are included in the article/Supplementary Materials. Further inquiries can be directed to the corresponding author.

## Supplementary Materials

The following supporting information can be downloaded at https://www.mdpi.com/article/10.3390/ph19091451/s1, Figure S1: Total ion chromatograms (TIC) in both positive and negative ion modes for UAE-DW, UAE-EtOH, ME-DW and ME-EtOH; Table S1: Phytochemical profiling of UAE-DW using LC-ESI-TOF-MS in positive ion mode; Table S2: Phytochemical profiling of UAE-EtOH using LC-ESI-TOF-MS in positive ion mode; Table S3: Phytochemical profiling of ME-DW using LC-ESI-TOF-MS in positive ion mode; Table S4: Phytochemical profiling of ME-EtOH using LC-ESI-TOF-MS in positive ion mode; Table S5: Phytochemical profiling of UAE-DW using LC-ESI-TOF-MS in negative ion mode; Table S6: Phytochemical profiling of UAE-EtOH using LC-ESI-TOF-MS in negative ion mode; Table S7: Phytochemical profiling of ME-DW using LC-ESI-TOF-MS in negative ion mode; Table S8: Phytochemical profiling of ME-EtOH using LC-ESI-TOF-MS in negative ion mode; Table S9: Participant demographic data.
