# Supplementary material for: In Vitro and Clinical Dermatological Effects of Rubus crataegifolius Fruit Extracts on Human Fibroblasts and Keratinocytes
Source: Pharmaceuticals (Basel). 2026 Sep 13;19(9):1451. doi: 10.3390/ph19091451 (PMC13610178; doi:10.3390/ph19091451)
Supplement: Supplementary file 1 [file pharmaceuticals-19-01451-s001.zip › pharmaceuticals-4512118-supplementary.pdf]

Supplementary material

(A)

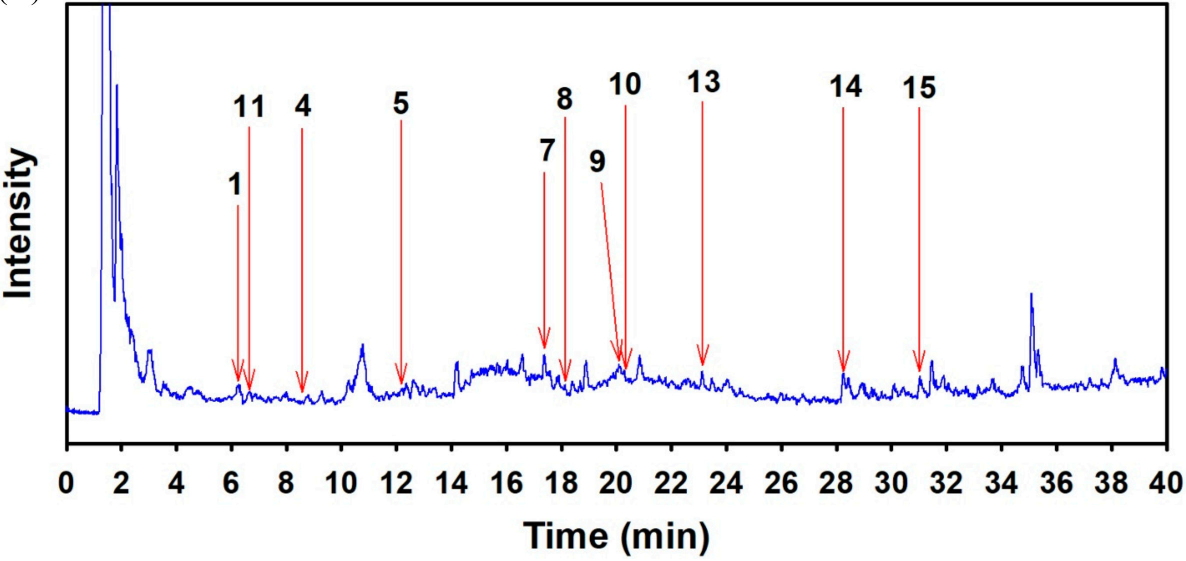

(B)

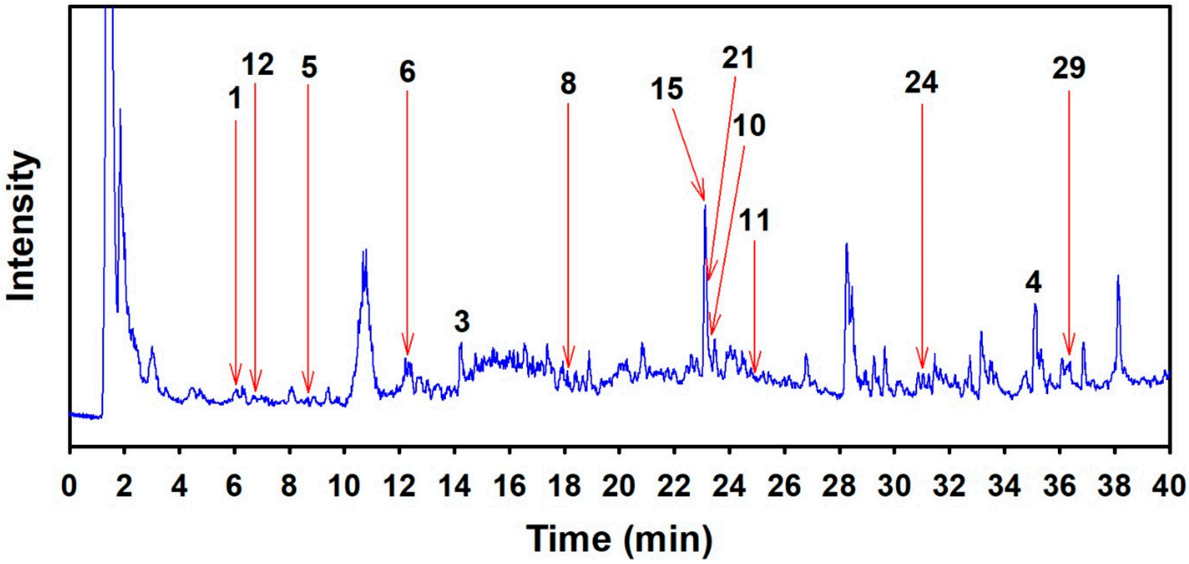

(C)

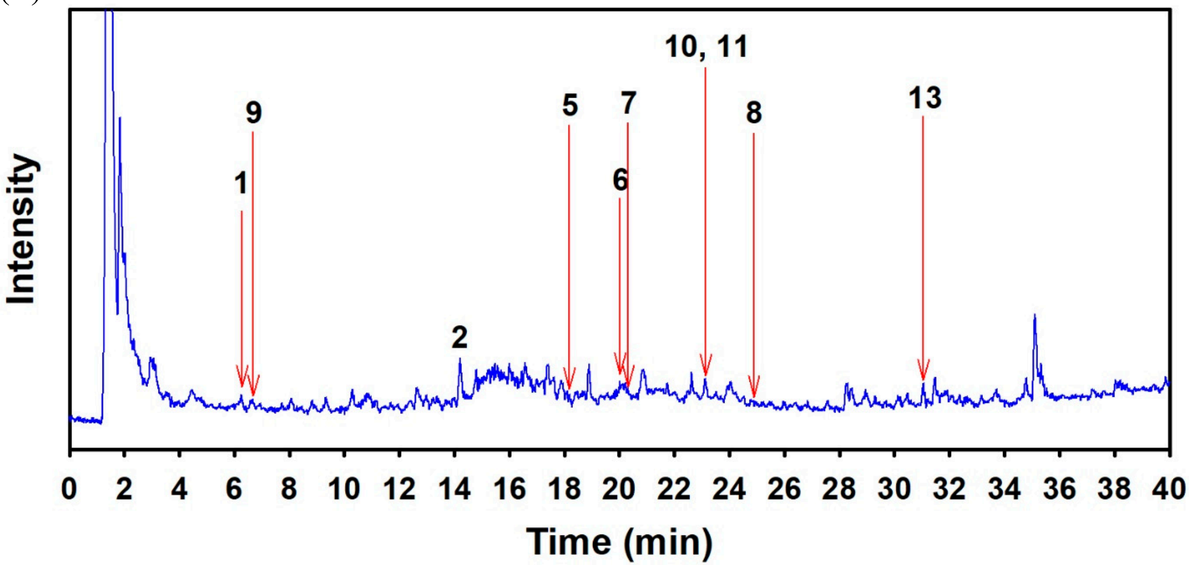

(D)

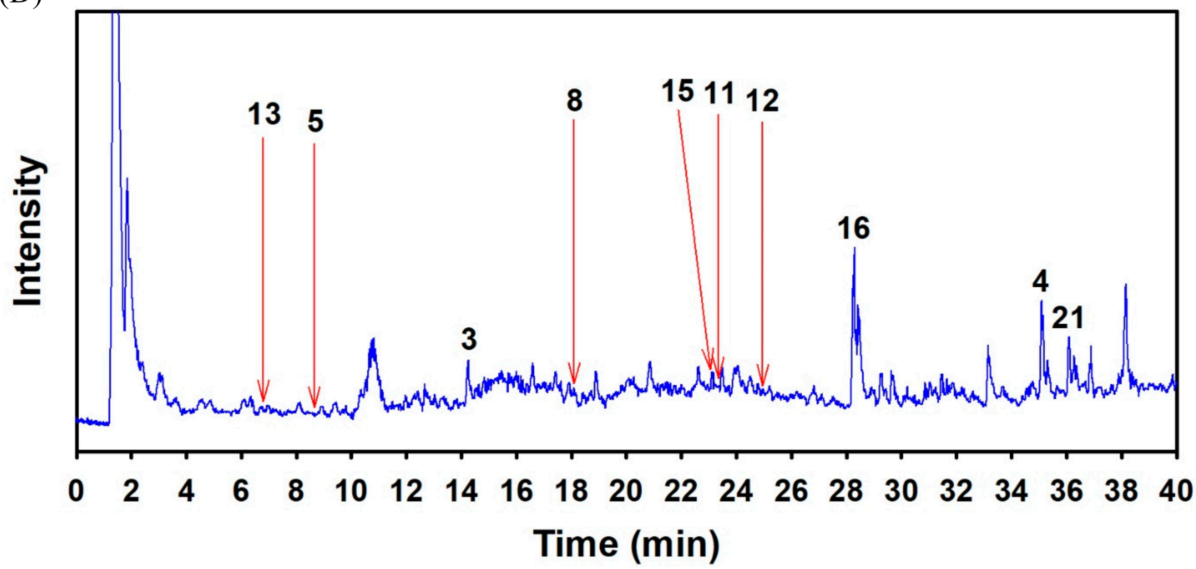
13
5
3
8
15
11
12
16
4
21
Intensity
0 2 4 6 8 10 12 14 16 18 20 22 24 26 28 30 32 34 36 38 40
Time (min)

(E)

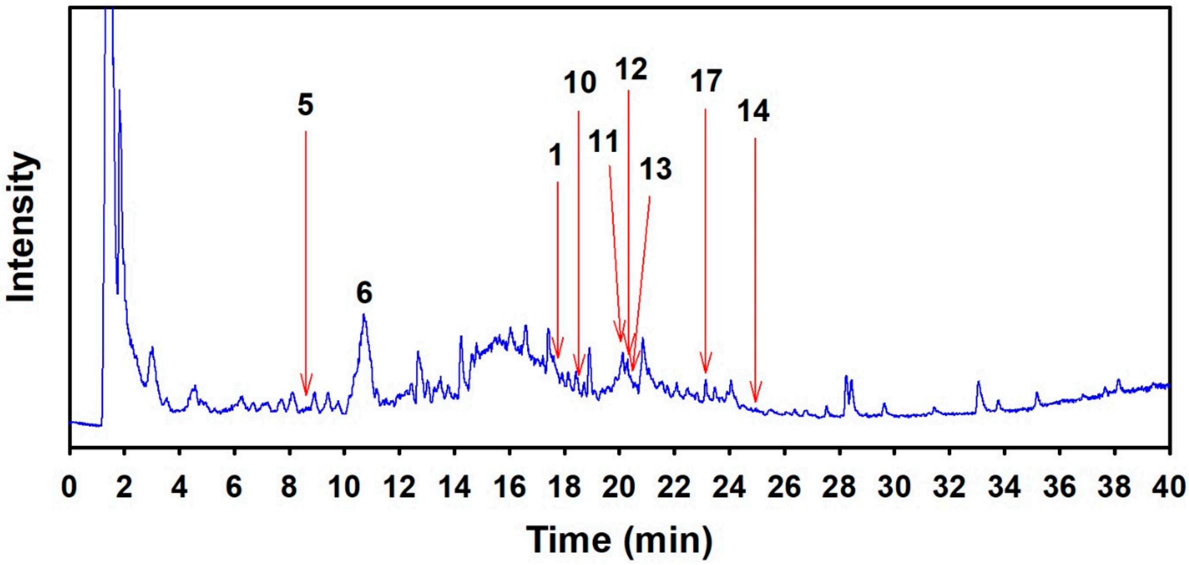
5
6
1
10
11
12
13
17
14
Intensity
0 2 4 6 8 10 12 14 16 18 20 22 24 26 28 30 32 34 36 38 40
Time (min)

(F)

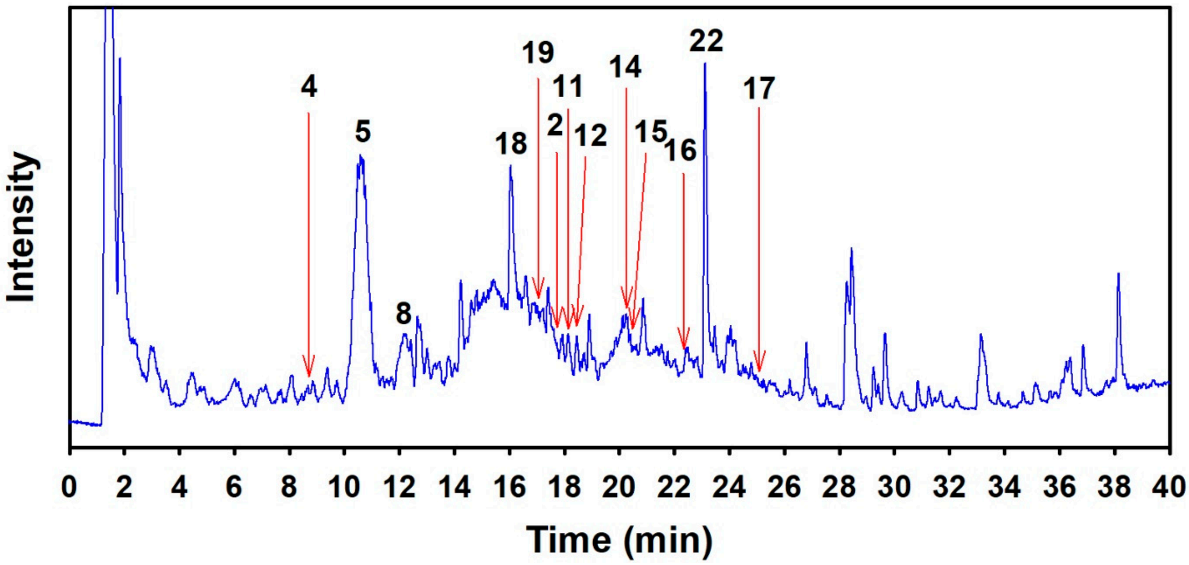
4
5
8
18
19
2
11
12
14
15
16
22
17
Intensity
0 2 4 6 8 10 12 14 16 18 20 22 24 26 28 30 32 34 36 38 40
Time (min)

(G)

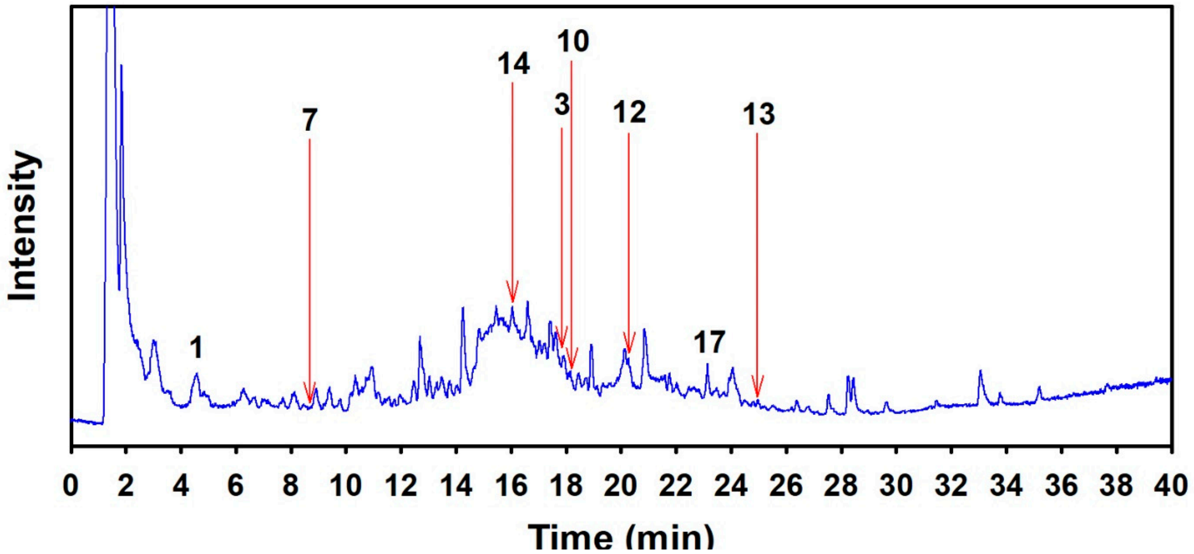

(H)

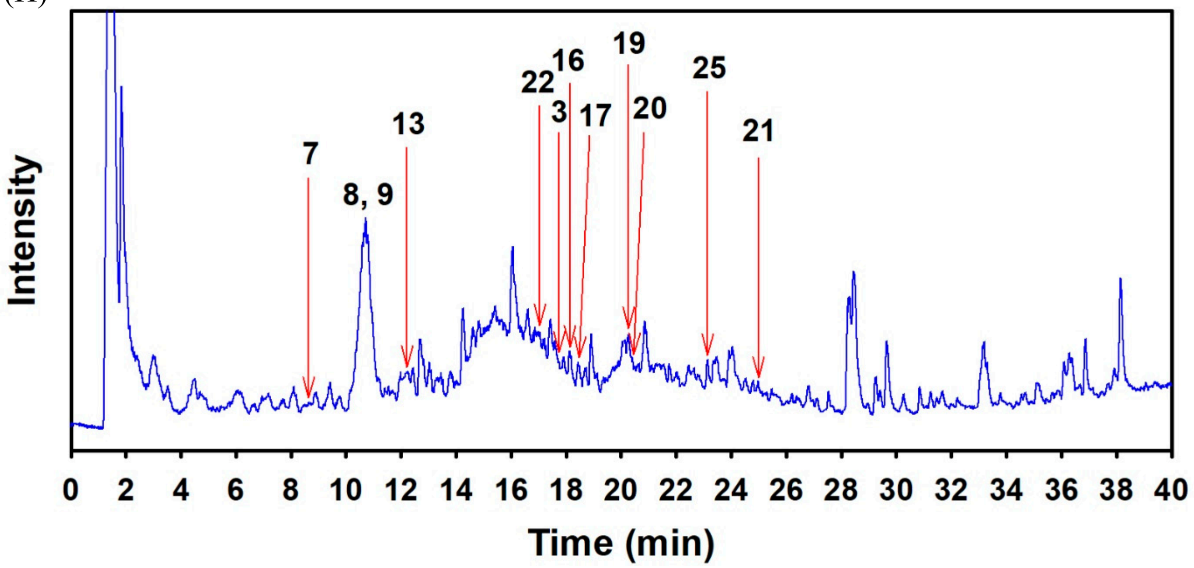

Figure S1. Total ion chromatograms (TIC) in both positive and negative ion modes for UAE-DW, UAE-EtOH, ME-DW and ME-EtOH. Each number in TIC corresponds to the number in Tables S1 ~ S8 with the corresponding compound shown in bold. (A) UAE-DW in positive ion mode (refer to Table S1), (B) UAE-EtOH in positive ion mode (refer to Table S2), (C) ME-DW in positive ion mode (refer to Table S3), (D) ME-EtOH in positive ion mode (refer to Table S4), (E) UAE-DW in negative ion mode (refer to Table S5), (F) UAE-EtOH in negative ion mode (refer to

Table S6), (G) ME-DW in negative ion mode (refer to Table S7), (H) ME-EtOH in negative ion mode (refer to Table S8).

Table S1. Phytochemical profiling of UAE-DW using LC-ESI-TOF-MS in positive ion mode

No.	retention time (min)	analyte name	molecular formula	molecular weight	theoretical mass (*m/z*)	observed mass (*m/z*)	adduct	error (ppm)
				organic acid				
**1**	**6.29**	**Indolelactic acid**	$C_{11}H_{11}NO_3$	**205.074**	**188.0699**	**188.0699**	**$[M+H-H_2O]^+$**	**0.0**
2	14.20	Glutaminyl-Proline	$C_{10}H_{17}N_3O_4$	243.122	243.1220	243.1221	$[M]^+$	0.2
				polyphenol (curcumin)				
3	35.11	Artonin C	$C_{40}H_{38}O_{10}$	678.246	679.2504	679.2508	$[M+H]^+$	0.6
				polyphenol (flavonoid)				
**4**	**8.54**	**Catechin**	$C_{15}H_{14}O_6$	**290.079**	**291.0861**	**291.0860**	**$[M+H]^+$**	**-0.2**
**5**	**12.25**	**Genistin**	$C_{21}H_{20}O_{10}$	**432.106**	**433.1125**	**433.1124**	**$[M+H]^+$**	**-0.1**
6	12.25	Prunin	$C_{21}H_{22}O_{10}$	434.121	434.1161	434.1169	$[M]^+$	1.8
**7**	**17.39**	**4-[5-(4-hydroxy-3-methoxyphenyl)-3,4-dimethyloxolan-2-yl]-2-methoxyphenol**	$C_{20}H_{24}O_5$	**344.162**	**327.1587**	**327.1588**	**$[M+H-H_2O]^+$**	**0.2**
**8**	**18.12**	**Quercetin glucuronide**	$C_{21}H_{18}O_{13}$	**478.075**	**479.0812**	**479.0813**	**$[M+H]^+$**	**0.1**
**9**	**20.04**	**Dihydrokaempferol**	$C_{15}H_{12}O_6$	**288.063**	**289.0706**	**289.0706**	**$[M+H]^+$**	**0.0**
**10**	**20.24**	**Quercetin**	$C_{15}H_{10}O_7$	**302.043**	**303.0496**	**303.0496**	**$[M+H]^+$**	**-0.3**
				polyphenol (phenolic acid)				
**11**	**6.66**	**Gallate**	$C_7H_6O_5$	**170.022**	**153.0177**	**153.0177**	**$[M+H-H_2O]^+$**	**0.2**
				polyphenol (tannin)				
12	16.85	3,4-Hexahydroxydiphenoylarabinose	$C_{19}H_{16}O_{13}$	452.059	435.0552	435.0550	$[M+H-H_2O]^+$	-0.5
				terpenoid				
**13**	**23.13**	**Abscisic acid**	$C_{15}H_{20}O_4$	**264.136**	**247.1322**	**247.1322**	**$[M+H-H_2O]^+$**	**0.1**
			$C_{15}H_{20}O_4$	**264.136**	**287.1251**	**287.1250**	**$[M+Na]^+$**	**-0.2**
**14**	**28.26**	**Lucyin A**	$C_{30}H_{46}O_5$	**486.335**	**469.3306**	**469.3304**	**$[M+H-H_2O]^+$**	**-0.3**

**15**	**31.05**	**Gingerenone B**	$C_{22}H_{26}O_6$	**386.173**	**387.1800**	**387.1801**	$[M+H]^+$	**0.3**

Table S2. Phytochemical profiling of UAE-EtOH using LC-ESI-TOF-MS in positive ion mode

No.	retention time (min)	analyte name	molecular formula	molecular weight	theoretical mass (*m/z*)	observed mass (*m/z*)	adduct	error (ppm)
				organic acid				
**1**	**6.08**	**3-Hydroxy-3-carboxymethyl-adipic acid**	$C_8H_{12}O_7$	**220.058**	**221.0649**	**221.0649**	$[M+H]^+$	**-0.1**
2	6.35	Indolelactic acid	$C_{11}H_{11}NO_3$	205.074	188.0697	188.0697	$[M+H-H_2O]^+$	-0.2
3	14.25	Glutaminyl-Proline	$C_{10}H_{17}N_3O_4$	243.122	243.1222	243.1221	$[M]^+$	-0.3
				polyphenol (curcumin)				
**4**	**35.12**	**Artonin C**	$C_{40}H_{38}O_{10}$	**678.246**	**679.2501**	**679.2504**	$[M+H]^+$	**0.3**
				polyphenol (flavonoid)				
**5**	**8.66**	**Catechin**	$C_{15}H_{14}O_6$	**290.079**	**291.0858**	**291.0858**	$[M+H]^+$	**0.1**
**6**	**12.29**	**Genistin**	$C_{21}H_{20}O_{10}$	**432.106**	**433.1120**	**433.1118**	$[M+H]^+$	**-0.5**
7	17.41	4-[5-(4-hydroxy-3-methoxyphenyl)-3,4-dimethyloxolan-2-yl]-2-methoxyphenol	$C_{20}H_{24}O_5$	344.162	327.1587	327.1587	$[M+H-H_2O]^+$	0.0
**8**	**18.14**	**Quercetin glucuronide**	$C_{21}H_{18}O_{13}$	**478.075**	**479.0813**	**479.0813**	$[M+H]^+$	**0.0**
9	20.57	3,3',4',5,6,7,8-Heptahydroxyflavone	$C_{15}H_{12}O_5$	334.032	317.0291	317.0291	$[M+H-H_2O]^+$	-0.1
**10**	**23.35**	**Quercetin**	$C_{15}H_{10}O_7$	**302.043**	**303.0500**	**303.0498**	$[M+H]^+$	**-0.3**
**11**	**24.95**	**Naringenin**	$C_{15}H_{12}O_5$	**272.068**	**273.0752**	**273.0753**	$[M+H]^+$	**0.3**
				polyphenol (phenolic acid)				
**12**	**6.72**	**Gallate**	$C_7H_6O_5$	**170.022**	**153.0176**	**153.0175**	$[M+H-H_2O]^+$	**-0.3**
				polyphenol (tannin)				
13	16.85	3,4-Hexahydroxydiphenoylarabinose	$C_{19}H_{16}O_{13}$	452.059	435.0551	435.0551	$[M+H-H_2O]^+$	0.0
				terpenoid				

14	23.12	NCGC00385328-01_C30H48O6_(1alpha,2alpha,3beta,5xi,9xi,18xi)-1,2,3,19-Tetrahydroxyurs-12-en-28-oic acid	$C_{30}H_{48}O_6$	504.345	505.3515	505.3515	$[M+H]^+$	0.0
**15**	**23.13**	**Abscisic acid**	$\mathbf{C_{15}H_{20}O_4}$	**264.136**	**247.1323**	**247.1323**	$\mathbf{[M+H-H_2O]^+}$	**-0.2**
16	23.13	NCGC00385328-01_C30H48O6_(1alpha,2alpha,3beta,5xi,9xi,18xi)-1,2,3,19-Tetrahydroxyurs-12-en-28-oic acid	$C_{30}H_{48}O_6$	504.345	487.3409	487.3408	$[M+H-H_2O]^+$	-0.2
17	23.13	Lucyin A	$C_{30}H_{46}O_5$	486.335	469.3304	469.3303	$[M+H-H_2O]^+$	-0.2
18	23.13	Glabrolide	$C_{30}H_{44}O_4$	468.324	451.3200	451.3198	$[M+H-H_2O]^+$	-0.3
19	23.13	2a-Hydroxygypsogenin 3-O-b-D-glucoside	$C_{30}H_{56}O_{10}$	648.387	631.3833	631.3832	$[M+H-H_2O]^+$	-0.1
20	23.13	2a-Hydroxygypsogenin 3-O-b-D-glucoside	$C_{30}H_{56}O_{10}$	648.387	649.3939	649.3936	$[M+H]^+$	-0.4
**21**	**23.14**	**Abscisic acid**	$\mathbf{C_{15}H_{20}O_4}$	**264.136**	**287.1249**	**287.1250**	$\mathbf{[M+Na]^+}$	**0.3**
22	28.28	Pygenic acid C	$C_{30}H_{48}O_6$	504.345	1009.6953	1009.6962	$[2M+H]^+$	0.8
23	28.29	Glabrolide	$C_{30}H_{44}O_4$	468.324	451.3196	451.3194	$[M+H-H_2O]^+$	-0.4
**24**	**31.05**	**Gingerenone B**	$\mathbf{C_{22}H_{26}O_6}$	**386.173**	**387.1797**	**387.1798**	$\mathbf{[M+H]^+}$	**0.2**
25	33.17	(1R,2R,4aS,6aS,6bR,10S,12aR,14bS)-1,8,10-trihydroxy-1,2,6a,6b,9,9,12a-heptamethyl-2,3,4,5,6,6a,7,8,8a,10,11,12,13,14b-tetradecahydropicene-4a-carboxylic acid	$C_{30}H_{48}O_5$	488.350	471.3460	471.3460	$[M+H-H_2O]^+$	0.0
26	33.18	(1R,2R,4aS,6aS,6bR,10S,12aR,14bS)-1,8,10-trihydroxy-1,2,6a,6b,9,9,12a-heptamethyl-2,3,4,5,6,6a,7,8,8a,10,11,12,13,14b-	$C_{30}H_{48}O_5$	488.350	489.3564	489.3566	$[M+H]^+$	0.3

		tetradecahydropicene-4a-carboxylic acid						
27	33.18	NCGC00179701-03_C30H46O4_Lup-20(29)-en-28-oic acid, 6-hydroxy-3-oxo-, (5xi,9xi,18xi)-	$C_{30}H_{46}O_4$	470.340	453.3354	453.3354	$[M+H-H_2O]^+$	-0.1
28	36.11	Erinacine D	$C_{27}H_{42}O_7$	478.293	478.2918	478.2917	$[M]^+$	-0.2
**29**	**36.39**	**Glabric acid**	$\mathbf{C_{30}H_{46}O_5}$	**486.335**	**487.3410**	**487.3407**	$\mathbf{[M+H]^+}$	**-0.5**
30	40.11	T2 Triol	$C_{20}H_{30}O_7$	382.199	383.2035	383.2036	$[M+H]^+$	0.3

Table S3. Phytochemical profiling of ME-DW using LC-ESI-TOF-MS in positive ion mode

No.	retention time (min)	analyte name	molecular formula	molecular weight	theoretical mass (*m/z*)	observed mass (*m/z*)	adduct	error (ppm)
				organic acid				
**1**	**6.28**	**Indolelactic acid**	$\mathbf{C_{11}H_{11}NO_3}$	**205.074**	**188.0699**	**188.0700**	$\mathbf{[M+H-H_2O]^+}$	**0.6**
**2**	**14.22**	**Glutaminyl-Proline**	$\mathbf{C_{10}H_{17}N_3O_4}$	**243.122**	**243.1222**	**243.1220**	$\mathbf{[M]^+}$	**-0.9**
				polyphenol (flavonoid)				
3	11.95	5-Hydroxy-3,3',7,8-tetramethoxy-4',5'-methylenedioxyflavone	$C_{20}H_{18}O_9$	402.095	403.1019	403.1018	$[M+H]^+$	-0.2
4	17.40	4-[5-(4-hydroxy-3-methoxyphenyl)-3,4-dimethyloxolan-2-yl]-2-methoxyphenol	$C_{20}H_{24}O_5$	344.162	327.1588	327.1587	$[M+H-H_2O]^+$	0.0
**5**	**18.14**	**Quercetin glucuronide**	$\mathbf{C_{21}H_{18}O_{13}}$	**478.075**	**479.0814**	**479.0813**	$\mathbf{[M+H]^+}$	**-0.2**
**6**	**20.05**	**Dihydrokaempferol**	$\mathbf{C_{15}H_{12}O_6}$	**288.063**	**289.0702**	**289.0701**	$\mathbf{[M+H]^+}$	**-0.2**
**7**	**20.24**	**Quercetin**	$\mathbf{C_{15}H_{10}O_7}$	**302.043**	**303.0496**	**303.0495**	$\mathbf{[M+H]^+}$	**-0.4**
**8**	**24.95**	**Naringenin**	$\mathbf{C_{15}H_{12}O_5}$	**272.068**	**273.0754**	**273.0756**	$\mathbf{[M+H]^+}$	**0.7**
				polyphenol (phenolic acid)				
**9**	**6.65**	**Gallate**	$\mathbf{C_7H_6O_5}$	**170.022**	**153.0174**	**153.0173**	$\mathbf{[M+H-H_2O]^+}$	**-0.7**
				terpenoid				
**10**	**23.13**	**Abscisic acid**	$\mathbf{C_{15}H_{20}O_4}$	**264.136**	**247.1322**	**247.1320**	$\mathbf{[M+H-H_2O]^+}$	**-0.8**
**11**	**23.14**	**Abscisic acid**	$\mathbf{C_{15}H_{20}O_4}$	**264.136**	**287.1252**	**287.1250**	$\mathbf{[M+Na]^+}$	**-0.4**
12	23.99	7-epi-Baccatin III	$C_{31}H_{38}O_{11}$	586.241	609.2293	609.2291	$[M+Na]^+$	-0.3
**13**	**31.05**	**Gingerenone B**	$\mathbf{C_{22}H_{26}O_6}$	**386.173**	**387.1801**	**387.1800**	$\mathbf{[M+H]^+}$	**-0.1**
14	40.12	T2 Triol	$C_{20}H_{30}O_7$	382.199	383.2035	383.2036	$[M+H]^+$	0.0

Table S4. Phytochemical profiling of ME-EtOH using LC-ESI-TOF-MS in positive ion mode

No.	retention time (min)	analyte name	molecular formula	molecular weight	theoretical mass (*m/z*)	observed mass (*m/z*)	adduct	error (ppm)
				organic acid				
1	6.12	3-Hydroxy-3-carboxymethyl-adipic acid	$C_8H_{12}O_7$	220.058	221.0648	221.0650	$[M+H]^+$	0.9
2	6.37	Indolelactic acid	$C_{11}H_{11}NO_3$	205.074	188.0696	188.0694	$[M+H-H_2O]^+$	-0.7
**3**	**14.22**	**Glutaminyl-Proline**	$C_{10}H_{17}N_3O_4$	**243.122**	**243.1219**	**243.1219**	$[M]^+$	**-0.3**
				polyphenol (curcumin)				
**4**	**35.12**	**Artonin C**	$C_{40}H_{38}O_{10}$	**678.246**	**679.2499**	**679.2503**	$[M+H]^+$	**0.6**
				polyphenol (flavonoid)				
**5**	**8.66**	**Catechin**	$C_{15}H_{14}O_6$	**290.079**	**291.0858**	**291.0859**	$[M+H]^+$	**0.2**
6	11.99	5-Hydroxy-3,3',7,8-tetramethoxy-4',5'-methylenedioxyflavone	$C_{20}H_{18}O_9$	402.095	403.1015	403.1016	$[M+H]^+$	0.1
7	17.42	4-[5-(4-hydroxy-3-methoxyphenyl)-3,4-dimethyloxolan-2-yl]-2-methoxyphenol	$C_{20}H_{24}O_5$	344.162	327.1586	327.1584	$[M+H-H_2O]^+$	-0.4
**8**	**18.14**	**Quercetin glucuronide**	$C_{21}H_{18}O_{13}$	**478.075**	**479.0812**	**479.0810**	$[M+H]^+$	**-0.4**
9	20.06	(2S,3S)-3,5,7-trihydroxy-2-(4-hydroxyphenyl)-2,3-dihydrochromen-4-one	$C_{15}H_{12}O_6$	288.063	289.0699	289.0698	$[M+H]^+$	-0.4
10	20.58	3,3',4',5,6,7,8-Heptahydroxyflavone	$C_{15}H_{10}O_9$	334.032	317.0288	317.0290	$[M+H-H_2O]^+$	0.5
**11**	**23.35**	**Quercetin**	$C_{15}H_{10}O_7$	**302.043**	**303.0497**	**303.0498**	$[M+H]^+$	**0.5**
**12**	**24.96**	**Naringenin**	$C_{15}H_{12}O_5$	**272.068**	**273.0753**	**273.0753**	$[M+H]^+$	**0.1**
				polyphenol (phenolic acid)				
**13**	**6.75**	**Gallate**	$C_7H_6O_5$	**170.022**	**153.0172**	**153.0174**	$[M+H-H_2O]^+$	**1.0**
				polyphenol (tannin)				
14	16.86	3,4-	$C_{19}H_{16}O_{13}$	452.059	435.0550	435.0549	$[M+H-H_2O]^+$	-0.2

		Hexahydroxydiphenoylarabinose						
				terpenoid				
**15**	**23.13**	**Abscisic acid**	**$C_{15}H_{20}O_4$**	**264.136**	**247.1323**	**247.1323**	**$[M+H-H_2O]^+$**	**0.1**
			$C_{15}H_{20}O_4$	264.136	287.1250	287.1250	$[M+Na]^+$	0.1
**16**	**28.28**	**Glabrolide**	**$C_{30}H_{44}O_4$**	**468.324**	**451.3196**	**451.3196**	**$[M+H-H_2O]^+$**	**0.1**
17	31.05	Gingerenone B	$C_{22}H_{26}O_6$	386.173	387.1796	387.1796	$[M+H]^+$	-0.1
18	33.18	(1R,2R,4aS,6aS,6bR,10S,12aR,14bS)-1,8,10-trihydroxy-1,2,6a,6b,9,9,12a-heptamethyl-2,3,4,5,6,6a,7,8,8a,10,11,12,13,14b-tetradecahydropicene-4a-carboxylic acid	$C_{30}H_{48}O_5$	488.350	471.3461	471.3461	$[M+H-H_2O]^+$	0.0
19	33.18	NCGC00179701-03_C30H46O4_Lup-20(29)-en-28-oic acid, 6-hydroxy-3-oxo-, (5xi,9xi,18xi)-	$C_{30}H_{46}O_4$	470.340	453.3355	453.3354	$[M+H-H_2O]^+$	-0.2
20	33.19	(1R,2R,4aS,6aS,6bR,10S,12aR,14bS)-1,8,10-trihydroxy-1,2,6a,6b,9,9,12a-heptamethyl-2,3,4,5,6,6a,7,8,8a,10,11,12,13,14b-tetradecahydropicene-4a-carboxylic acid	$C_{30}H_{48}O_5$	488.350	489.3565	489.3564	$[M+H]^+$	-0.1
**21**	**36.10**	**Erinacine D**	**$C_{27}H_{42}O_7$**	**478.293**	**478.2917**	**478.2916**	**$[M]^+$**	**-0.2**
22	36.40	Glabric acid	$C_{30}H_{46}O_5$	486.335	487.3406	487.3407	$[M+H]^+$	0.0

Table S5. Phytochemical profiling of UAE-DW using LC-ESI-TOF-MS in negative ion mode

No.	retention time (min)	analyte name	molecular formula	molecular weight	theoretical mass (*m/z*)	observed mass (*m/z*)	adduct	error (ppm)
				organic acid				
**1**	**17.75**	**Salicylic acid**	$\mathbf{C_7H_6O_3}$	**138.032**	**137.0254**	**137.0254**	**[M-H]$^-$**	**0.4**
2	23.38	5-(3',5'-Dihydroxyphenyl)-gamma-valerolactone-O-sulphate-O-methyl	$C_{12}H_{14}O_7S$	302.046	301.0357	301.0357	[M-H]$^-$	0.1
3	33.06	O-Benzyl-L-serine	$C_{10}H_{13}NO_3$	195.090	194.0829	194.0831	[M-H]$^-$	0.9
				polyphenol (flavonoid)				
4	6.34	Dihydromyricetin	$C_{15}H_{12}O_8$	320.053	319.0461	319.0461	[M-H]$^-$	0.0
**5**	**8.68**	**Catechin**	$\mathbf{C_{15}H_{14}O_6}$	**290.079**	**289.0723**	**289.0723**	**[M-H]$^-$**	**0.1**
**6**	**10.72**	**Cyanidin-3-O-glucoside**	$\mathbf{C_{21}H_{20}O_{11}}$	**448.101**	**447.0932**	**447.0934**	**[M-H]$^-$**	**0.4**
7	10.73	(-)-Epicatechin 3'-O-glucuronide	$C_{21}H_{22}O_{12}$	466.111	511.1079	511.1078	[M+HCO$_2$]$^-$	-0.1
8	13.02	(7'R,8'R)-4,7'-Epoxy-3'-methoxy-4',5,9,9'-lignanetetrol 9'-glucoside	$C_{25}H_{32}O_{11}$	508.194	553.1931	553.1932	[M+HCO$_2$]$^-$	0.4
9	14.61	3,5-dihydroxy-2-(4-hydroxyphenyl)-7-[3,4,5-trihydroxy-6-(hydroxymethyl)oxan-2-yl]oxy-2,3-dihydrochromen-4-one	$C_{21}H_{22}O_{11}$	450.116	449.1089	449.1091	[M-H]$^-$	0.3
**10**	**18.45**	**Hirsutrin**	$\mathbf{C_{21}H_{20}O_{12}}$	**464.095**	**463.0883**	**463.0882**	**[M-H]$^-$**	**-0.1**
**11**	**20.09**	**Dihydrokaempferol**	$\mathbf{C_{15}H_{12}O_6}$	**288.063**	**287.0568**	**287.0569**	**[M-H]$^-$**	**0.2**
**12**	**20.27**	**Quercetrin**	$\mathbf{C_{21}H_{20}O_{11}}$	**448.101**	**447.0936**	**447.0935**	**[M-H]$^-$**	**0.0**
**13**	**20.42**	**Naringenin-7-O-glucoside**	$\mathbf{C_{21}H_{22}O_{10}}$	**434.121**	**433.1142**	**433.1142**	**[M-H]$^-$**	**-0.1**
**14**	**24.97**	**Naringenin**	$\mathbf{C_{15}H_{12}O_5}$	**272.068**	**271.0617**	**271.0618**	**[M-H]$^-$**	**0.2**
				terpenoid				

15	12.71	Phaseolus e	$C_{25}H_{34}O_{12}$	526.205	571.2038	571.2037	$[M+HCO_2]^-$	-0.1
16	12.72	Phaseolus e	$C_{25}H_{34}O_{12}$	526.205	525.1980	525.1980	$[M-H]^-$	0.1
**17**	**23.14**	**Abscisic acid**	$\mathbf{C_{15}H_{20}O_4}$	**264.136**	**263.1294**	**263.1295**	$\mathbf{[M-H]^-}$	**0.3**
18	24.06	(3beta,17alpha,23S)-17,23-Epoxy-3,28,29-trihydroxy-27-norlanost-8-en-24-one	$C_{29}H_{46}O_5$	474.335	519.3326	519.3326	$[M+HCO_2]^-$	-0.1
19	28.26	Pygenic acid C	$C_{30}H_{48}O_6$	504.345	503.3384	503.3384	$[M-H]^-$	0.0
20	28.26	Madecassic acid	$C_{30}H_{48}O_6$	504.345	549.3440	549.3443	$[M+HCO_2]^-$	0.5

Table S6. Phytochemical profiling of UAE-EtOH using LC-ESI-TOF-MS in negative ion mode

No.	retention time (min)	analyte name	molecular formula	molecular weight	theoretical mass (*m/z*)	observed mass (*m/z*)	adduct	error (ppm)
				organic acid				
1	6.15	4-Ketopimelic acid	$C_7H_{10}O_5$	174.053	219.0515	219.0516	$[M-H]^-$	0.2
**2**	**17.74**	**Salicylic acid**	$C_7H_6O_3$	**138.032**	**137.0254**	**137.0254**	$[M-H]^-$	**0.3**
3	33.06	O-Benzyl-L-serine	$C_{10}H_{13}NO_3$	195.090	194.0830	194.0829	$[M-H]^-$	-0.8
				polyphenol (flavonoid)				
**4**	**8.66**	**Catechin**	$C_{15}H_{14}O_6$	**290.079**	**289.0722**	**289.0722**	$[M-H]^-$	**0.0**
**5**	**10.60**	**Cyanidin-3-O-glucoside**	$C_{21}H_{20}O_{11}$	**448.101**	**447.0931**	**447.0932**	$[M-H]^-$	**0.2**
			$C_{21}H_{20}O_{11}$	**448.101**	**895.1954**	**895.1957**	$[2M-H]^-$	**0.3**
6	10.60	(-)-Epicatechin 3'-O-glucuronide	$C_{21}H_{22}O_{12}$	466.111	511.1079	511.1083	$[M+HCO_2]^-$	0.9
7	10.68	NCGC00385782-01 (2R,3R)-2-(3,4-dihydroxyphenyl)-3,5,7-trihydroxy-6-[(3R,4R,5S,6R)-3,4,5-trihydroxy-6-(hydroxymethyl)oxan-2-yl]-2,3-dihydrochromen-4-one	$C_{21}H_{22}O_{12}$	466.111	465.1037	465.1038	$[M-H]^-$	0.2
**8**	**12.18**	**Genistin**	$C_{21}H_{20}O_{10}$	**432.106**	**431.0982**	**431.0982**	$[M-H]^-$	**-0.1**
9	13.00	(7'R,8'R)-4,7'-Epoxy-3'-methoxy-4',5,9,9'-lignanetetrol 9'-glucoside	$C_{25}H_{32}O_{11}$	508.194	553.1925	553.1925	$[M+HCO_2]^-$	0.0
10	14.61	3,5-dihydroxy-2-(4-hydroxyphenyl)-7-[3,4,5-trihydroxy-6-(hydroxymethyl)oxan-2-yl]oxy-2,3-dihydrochromen-4-one	$C_{21}H_{22}O_{11}$	450.116	449.1089	449.1089	$[M-H]^-$	0.1

**11**	**18.16**	**Quercetin glucuronide**	**$C_{21}H_{18}O_{13}$**	**478.075**	**477.0673**	**477.0673**	**$[M-H]^-$**	**0.0**
**12**	**18.46**	**Hirsutrin**	**$C_{21}H_{20}O_{12}$**	**464.095**	**463.0882**	**463.0882**	**$[M-H]^-$**	**0.1**
13	20.09	(2S,3S)-3,5,7-trihydroxy-2-(4-hydroxyphenyl)-2,3-dihydrochromen-4-one	$C_{15}H_{12}O_6$	288.063	287.0566	287.0566	$[M-H]^-$	0.2
**14**	**20.27**	**Quercetrin**	**$C_{21}H_{20}O_{11}$**	**448.101**	**447.0930**	**447.0930**	**$[M-H]^-$**	**0.2**
**15**	**20.41**	**Naringenin-7-O-glucoside**	**$C_{21}H_{22}O_{10}$**	**434.121**	**433.1138**	**433.1138**	**$[M-H]^-$**	**0.0**
**16**	**22.40**	**Quercetin**	**$C_{15}H_{10}O_7$**	**302.043**	**301.0356**	**301.0355**	**$[M-H]^-$**	**-0.3**
**17**	**24.98**	**Naringenin**	**$C_{15}H_{12}O_5$**	**272.068**	**271.0616**	**271.0616**	**$[M-H]^-$**	**0.2**
polyphenol (lignan)								
**18**	**16.03**	**Secoisolariciresinol diglucoside**	**$C_{32}H_{46}O_{16}$**	**686.279**	**731.2780**	**731.2779**	**$[M+HCO_2]^-$**	**-0.1**
polyphenol (phenolic acid)								
**19**	**17.05**	**Ellagic acid**	**$C_{14}H_6O_8$**	**302.006**	**300.9992**	**300.9991**	**$[M-H]^-$**	**-0.4**
terpenoid								
20	12.70	Phaseolus e	$C_{25}H_{34}O_{12}$	526.205	571.2035	571.2033	$[M+HCO_2]^-$	-0.2
21	12.71	Phaseolus e	$C_{25}H_{34}O_{12}$	526.205	525.1978	525.1978	$[M-H]^-$	0.2
**22**	**23.15**	**Abscisic acid**	**$C_{15}H_{20}O_4$**	**264.136**	**263.1290**	**263.1290**	**$[M-H]^-$**	**0.2**
23	23.99	Nimbin	$C_{30}H_{36}O_9$	540.236	585.2334	585.2335	$[M+HCO_2]^-$	0.2

Table S7. Phytochemical profiling of ME-DW using LC-ESI-TOF-MS in negative ion mode

No.	retention time (min)	analyte name	molecular formula	molecular weight	theoretical mass (*m/z*)	observed mass (*m/z*)	adduct	error (ppm)
				organic acid				
**1**	**4.57**	**Protocatehuic acid**	$\mathbf{C_7H_6O_4}$	**154.027**	**153.0192**	**153.0201**	**[M-H]$^-$**	**5.7**
2	8.10	2-Isopropylmalate	$C_7H_{12}O_5$	176.068	175.0621	175.0621	[M-H]$^-$	-0.1
**3**	**17.76**	**Salicylic acid**	$\mathbf{C_7H_6O_3}$	**138.032**	**137.0253**	**137.0253**	**[M-H]$^-$**	**0.0**
4	23.38	5-(3',5'-Dihydroxyphenyl)-gamma-valerolactone-O-sulphate-O-methyl	$C_{12}H_{14}O_7S$	302.046	301.0358	301.0359	[M-H]$^-$	0.2
5	33.06	O-Benzyl-L-serine	$C_{10}H_{13}NO_3$	195.090	194.0831	194.0831	[M-H]$^-$	0.3
				polyphenol (flavonoid)				
6	6.34	Dihydromyricetin	$C_{15}H_{12}O_8$	320.053	319.0465	319.0466	[M-H]$^-$	0.4
**7**	**8.69**	**Catechin**	$\mathbf{C_{15}H_{14}O_6}$	**290.079**	**289.0722**	**289.0724**	**[M-H]$^-$**	**0.6**
8	13.03	(7'R,8'R)-4,7'-Epoxy-3'-methoxy-4',5,9,9'-lignanetetrol 9'-glucoside	$C_{25}H_{32}O_{11}$	508.194	553.1936	553.1936	[M+HCO$_2$]$^-$	0.0
9	14.62	3,5-dihydroxy-2-(4-hydroxyphenyl)-7-[3,4,5-trihydroxy-6-(hydroxymethyl)oxan-2-yl]oxy-2,3-dihydrochromen-4-one	$C_{21}H_{22}O_{11}$	450.116	449.1095	449.1096	[M-H]$^-$	0.3
**10**	**18.16**	**Quercetin glucuronide**	$\mathbf{C_{21}H_{18}O_{13}}$	**478.075**	**477.0680**	**477.0682**	**[M-H]$^-$**	**0.4**
11	20.09	(2S,3S)-3,5,7-trihydroxy-2-(4-hydroxyphenyl)-2,3-dihydrochromen-4-one	$C_{15}H_{12}O_6$	288.063	287.0567	287.0565	[M-H]$^-$	-0.6
**12**	**20.27**	**Quercetrin**	$\mathbf{C_{21}H_{20}O_{11}}$	**448.101**	**447.0937**	**447.0937**	**[M-H]$^-$**	**0.0**
**13**	**24.98**	**Naringenin**	$\mathbf{C_{15}H_{12}O_5}$	**272.068**	**271.0618**	**271.0618**	**[M-H]$^-$**	**-0.1**
				polyphenol (lignan)				
**14**	**16.04**	**Secoisolariciresinol diglucoside**	$\mathbf{C_{32}H_{46}O_{16}}$	**686.279**	**731.2784**	**731.2789**	**[M+HCO$_2$]$^-$**	**0.6**

				terpenoid				
15	12.71	Phaseolus e	$C_{25}H_{34}O_{12}$	526.205	571.2040	571.2039	$[M+HCO_2]^-$	-0.2
16	12.72	Phaseolus e	$C_{25}H_{34}O_{12}$	526.205	525.1986	525.1985	$[M-H]^-$	-0.3
**17**	**23.14**	**Abscisic acid**	$\mathbf{C_{15}H_{20}O_4}$	**264.136**	**263.1296**	**263.1296**	$\mathbf{[M-H]^-}$	**-0.2**
18	28.26	Pygenic acid C	$C_{30}H_{48}O_6$	504.345	503.3383	503.3381	$[M-H]^-$	-0.3
19	28.26	Madecassic acid	$C_{30}H_{48}O_6$	504.345	549.3440	549.3438	$[M+HCO_2]^-$	-0.5

Table S8. Phytochemical profiling of ME-EtOH using LC-ESI-TOF-MS in negative ion mode

No.	retention time (min)	analyte name	molecular formula	molecular weight	theoretical mass (*m/z*)	observed mass (*m/z*)	adduct	error (ppm)
				organic acid				
1	6.17	4-Ketopimelic acid	$C_7H_{10}O_5$	174.100	219.0518	219.0517	$[M-H]^-$	-0.1
2	14.25	Prolyl-Glutamate	$C_{10}H_{15}N_2O_5$	243.098	485.1783	485.1884	$[2M-H]^-$	20.7
**3**	**17.74**	**Salicylic acid**	$\mathbf{C_7H_6O_3}$	**138.032**	**137.0256**	**137.0255**	$\mathbf{[M-H]^-}$	**-0.5**
4	23.37	5-(3',5'-Dihydroxyphenyl)-gamma-valerolactone-O-sulphate-O-methyl	$C_{12}H_{14}O_7S$	302.046	301.0363	301.0363	$[M-H]^-$	0.0
5	33.07	O-Benzyl-L-serine	$C_{10}H_{13}NO_3$	195.090	194.0832	194.0832	$[M-H]^-$	0.0
				polyphenol (coumarin)				
6	24.97	Oroselone	$C_{14}H_{10}O_3$	226.063	271.0621	271.0621	$[M+HCO_2]^-$	0.0
				polyphenol (flavonoid)				
**7**	**8.68**	**Catechin**	$\mathbf{C_{15}H_{14}O_6}$	**290.079**	**289.0723**	**289.0723**	$\mathbf{[M-H]^-}$	**-0.2**
**8**	**10.69**	**Luteolin 4'-glucoside**	$\mathbf{C_{21}H_{20}O_{11}}$	**448.101**	**447.0941**	**447.0939**	$\mathbf{[M-H]^-}$	**-0.3**
**9**	**10.70**	**Cyanidin-3-O-glucoside**	$\mathbf{C_{21}H_{20}O_{11}}$	**448.101**	**895.1972**	**895.1975**	$\mathbf{[2M-H]^-}$	**0.4**
10	10.70	NCGC00385782-01 (2R,3R)-2-(3,4-dihydroxyphenyl)-3,5,7-trihydroxy-6-[(3R,4R,5S,6R)-3,4,5-trihydroxy-6-(hydroxymethyl)oxan-2-yl]-2,3-dihydrochromen-4-one	$C_{21}H_{22}O_{12}$	466.111	465.1047	465.1045	$[M-H]^-$	-0.3
11	10.70	(-)-Epicatechin 3'-O-glucuronide	$C_{21}H_{22}O_{12}$	466.111	511.1087	511.1085	$[M+HCO_2]^-$	-0.4
12	11.99	5-Hydroxy-3,3',7,8-tetramethoxy-4',5'-methylenedioxyflavone	$C_{20}H_{18}O_9$	402.095	401.0883	401.0884	$[M-H]^-$	0.3

**13**	**12.20**	**Genistin**	**$C_{21}H_{20}O_{10}$**	**432.106**	**431.0988**	**431.0989**	**$[M-H]^-$**	**0.1**
14	13.02	(7'R,8'R)-4,7'-Epoxy-3'-methoxy-4',5,9,9'-lignanetetrol 9'-glucoside	$C_{25}H_{32}O_{11}$	508.194	553.1938	553.1939	$[M+HCO_2]^-$	0.2
15	14.60	3,5-dihydroxy-2-(4-hydroxyphenyl)-7-[3,4,5-trihydroxy-6-(hydroxymethyl)oxan-2-yl]oxy-2,3-dihydrochromen-4-one	$C_{21}H_{22}O_{11}$	450.116	449.1095	449.1095	$[M-H]^-$	0.2
**16**	**18.15**	**Quercetin glucuronide**	**$C_{21}H_{18}O_{13}$**	**478.075**	**477.0684**	**477.0684**	**$[M-H]^-$**	**0.0**
**17**	**18.45**	**Hirsutrin**	**$C_{21}H_{20}O_{12}$**	**464.095**	**463.0887**	**463.0890**	**$[M-H]^-$**	**0.6**
18	20.08	(2S,3S)-3,5,7-trihydroxy-2-(4-hydroxyphenyl)-2,3-dihydrochromen-4-one	$C_{15}H_{12}O_6$	288.063	287.0572	287.0574	$[M-H]^-$	0.7
**19**	**20.26**	**Quercetrin**	**$C_{21}H_{20}O_{11}$**	**448.101**	**447.0939**	**447.0939**	**$[M-H]^-$**	**0.1**
**20**	**20.41**	**Naringenin-7-O-glucoside**	**$C_{21}H_{22}O_{10}$**	**434.121**	**433.1143**	**433.1146**	**$[M-H]^-$**	**0.8**
**21**	**24.97**	**Naringenin**	**$C_{15}H_{12}O_5$**	**272.068**	**271.0621**	**271.0621**	**$[M-H]^-$**	**0.0**
				polyphenol (phenolic acid)				
**22**	**17.04**	**Ellagic acid**	**$C_{14}H_6O_8$**	**302.006**	**300.9999**	**300.9999**	**$[M-H]^-$**	**0.2**
				terpenoid				
23	12.70	Phaseolus e	$C_{25}H_{34}O_{12}$	526.205	571.2040	571.2041	$[M+HCO_2]^-$	0.1
24	12.71	Phaseolus e	$C_{25}H_{34}O_{12}$	526.205	525.1986	525.1985	$[M-H]^-$	-0.3
**25**	**23.15**	**Abscisic acid**	**$C_{15}H_{20}O_4$**	**264.136**	**263.1301**	**263.1300**	**$[M-H]^-$**	**-0.3**
26	23.99	Nimbin	$C_{30}H_{36}O_9$	540.236	585.2347	585.2354	$[M+HCO_2]^-$	1.1

Table S9. Participant demographic data

No.	Sex	Age	Adverse events
S01	F	58	None
S02	F	42	None
S03	F	41	None
S04	F	45	None
S05	F	59	None
S06	F	48	None
S07	F	48	None
S08	F	45	None
S09	F	45	None
S10	F	43	None
S11	F	45	None
S12	F	38	None
